# Quantitative and systematic behavioral profiling reveals social complexity in eusocial naked mole-rats

**DOI:** 10.1126/sciadv.ady0481

**Published:** 2025-10-08

**Authors:** Masanori Yamakawa, Takahiro Ezaki, Akiyuki Watarai, Nobuyuki Kutsukake, Kyoko Miura, Teruhiro Okuyama

**Affiliations:** ^1^ Department of Aging and Longevity Research, Faculty of Life Sciences, Kumamoto University, Kumamoto, Japan.; ^2^ Department of Evolutionary Studies of Biosystems, The Graduate University for Advanced Studies, SOKENDAI, Hayama, Kanagawa, Japan.; ^3^ Institute for Quantitative Biosciences, The University of Tokyo, Tokyo, Japan.; ^4^ Research Center for Advanced Science and Technology, The University of Tokyo, Tokyo, Japan.; ^5^ Research Center for Integrative Evolutionary Science, The Graduate University for Advanced Studies, SOKENDAI, Hayama, Kanagawa, Japan.; ^6^ Laboratory of Longevity and Stem Cell Biology, Department of Stem Cell Biology and Medicine, Graduate School of Medical Sciences, Kyushu University, Fukuoka, Japan.; ^7^ Center for Metabolic Regulation of Healthy Aging, Kumamoto University, Kumamoto, Japan.

## Abstract

In highly organized animal societies, individual behavioral differences and close social relationships are crucial for group success and cohesion. However, in naked mole-rats, a eusocial mammal, these factors remain poorly understood because monitoring all colony members simultaneously is challenging. We developed an automated radio frequency identification (RFID) tracking system to continuously collect behavioral data from entire colonies, monitoring 102 individuals from five colonies for 30 days. Based on behavioral parameters, we statistically identified distinct behavioral phenotypes, comprising one cluster for breeders and six clusters for nonbreeders. Breeders formed strong social bonds, consistently remaining close in activity rhythm synchrony, spatial proximity, and directional following. In contrast, nonbreeders exhibited behavioral heterogeneity according to their cluster: One cluster avoided other active nonbreeders, whereas another cluster attracted frequent following. Our study highlights social complexity in this eusocial mammal and establishes a robust platform for further investigations into naked mole-rat social dynamics.

## Teaser

Automated tracking in naked mole-rats reveals behavioral specificity of breeders and diversity of nonbreeders in a colony.

## INTRODUCTION

In highly organized societies, the success and stability of a group often rely on the behaviors of individuals and their interactions. For example, behavioral individual differences within a group can lead to task allocation, which enhances efficiency in group task performance (*1*–*5*). In addition, close social interactions and relationships further contribute to effective and resilient group maintenance (*6*–*9*). Therefore, to understand the success and stability of such cooperative societies, it is crucial to investigate individual behavioral differences and the nature of social relationships. Notably, recent advances in technology have enabled efficient and automated methods for recording the behaviors of multiple individuals simultaneously (*10*–*13*). Especially in eusocial insects, long-term behavioral data collection by automated tracking has revealed previously undetectable individual differences and social relationships (*10*, *14*). Similarly, in laboratory animals such as mice, long-term research has been accomplished by developing an automated method of recording social behavior of multiple individuals simultaneously (*15*, *16*).

The naked mole-rat (*Heterocephalus glaber*), a eusocial mammal, forms highly organized cooperative societies (*17*). They form a single-family group (colony) consisting of several dozen individuals, with a clear reproductive division of labor: Only one breeding female and one to three breeding males reproduce, whereas nonbreeders are physiologically inhibited in reproduction and perform various tasks (*17*–*19*). Despite extensive research, the following two major aspects of naked mole-rat societies are still poorly understood. First, the composition of behavioral phenotypes in a steady-state colony is unclear. Early studies classified individuals into three categories based on activity levels: “frequent workers,” “infrequent workers,” and “non-workers” (*17*). However, later studies suggested that nonbreeders exhibit a continuum of behavioral traits across various measures (*20*). In addition, task allocation systems have been investigated with a focus on the division of labor. For instance, task specialization has been suggested, with larger or older individuals assigned to opportunistic defense against conspecific intruders or predators invading the burrow, whereas smaller or younger individuals typically handle routine tasks (*20*–*22*). On the other hand, task allocation systems for daily activities and the effects of associated individual attribute factors vary widely among colonies and laboratories (*20*–*26*). Given the above, a general framework for understanding behavioral phenotypes in steady-state colonies has yet to be established. Second, the nature of social relationships within colonies is largely unexplored. Previous studies have noted variations in aggression from breeders (especially breeding females) toward nonbreeders in terms of frequency and degree (*27*, *28*). However, there is little information on how close social relationships are distributed throughout a colony. Social relationships depend not only on direct interactions but also on spatiotemporal distribution patterns within a colony, which have not been systematically studied in naked mole-rats.

These gaps in knowledge are partly due to the limitations of previous studies of naked mole-rats, such as small datasets and low-resolution behavioral observations caused by the manual approach to behavioral recording. The efficient methods of behavioral recording have not been developed in this research field, and there are no studies utilizing large-scale behavior analysis. To address these challenges, we developed a long-term automated tracking system using a radio frequency identification (RFID) technology to monitor all individuals in a colony. Using this system, we collected large-scale behavioral data and investigated two main topics: (i) the composition of behavioral phenotypes within colonies, determined through clustering of multidimensional behaviors, and (ii) the nature of social relationships across all dyads, analyzed from three perspectives: activity rhythm synchrony, spatial proximity, and following frequency.

## RESULTS

### Development of an RFID-based automated tracking system

**Fig. 1.**
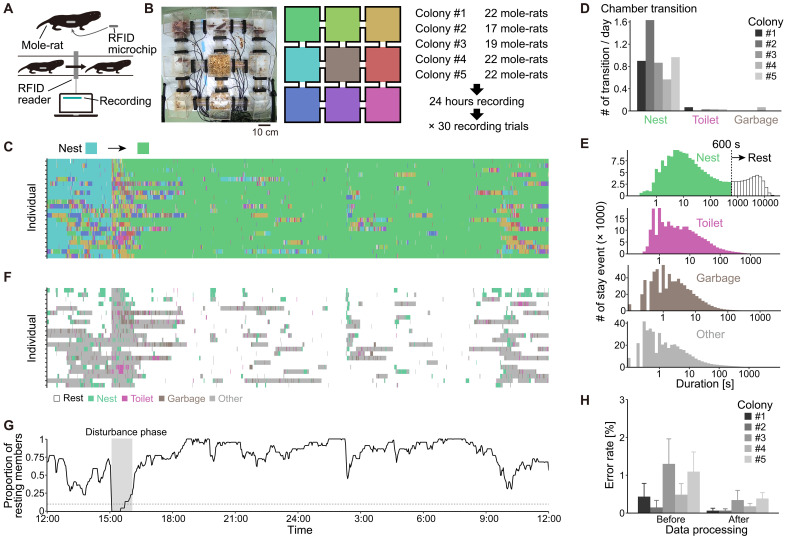
An RFID-based automated tracking system. (**A**) Setup for tracking individuals using RFID technology. (**B**) Structure of the experimental setup and overview of the data collection. Left: A top-down photograph of the housing system. Right: A color-coded layout of chambers and the data collection workflow. (**C**) Example of 1-day time-series location data for each individual in colony #1. Colors correspond to chamber IDs in (B). The nest, defined by majority occupancy, shifted once during the day. (**D**) Mean number of daily nest, toilet, and garbage location transitions per colony. (**E**) Frequency distribution of stay durations in nest, toilet, garbage, and other areas. (**F**) Time-series behavioral data for each individual in colony #1 on the same day as in (C). (**G**) Time-series of the proportion of resting members in colony #1 on the same day. The *x* axis is shared with [(C) and (F)]. Disturbance phases (<25% resting with a dip below 10%, dashed line) were excluded from analysis. (**H**) Error rates (means ± SD) representing failure probabilities of individual localization, before and after data processing, for each colony.

To simultaneously monitor the long-term movements of all colony members, we developed an automated multiple-animal tracking system using RFID technology (Fig. 1A). Compared to image-based systems, our RFID-based approach is better suited for naked mole-rats, as it allows large-scale data collection (i.e., multiple individuals over extended periods) and accommodates the animals’ tendency to frequently overlap with one another during both active and resting states. To streamline analyses, we used a straightforward housing design: nine acrylic boxes (15 cm by 15 cm by 20 cm in height) arranged in a square on a flat surface, each connected to its neighbors by acrylic pipes (Fig. 1B). This configuration produced a five-by-five grid of compartments delineated by the boxes and pipes. We positioned 24 RFID readers (each with a 6-cm-diameter ring-shaped antenna) at both ends of the pipes. Each subject animal received a subcutaneous RFID microchip implant (2.1 mm in diameter by 12 mm in length, 0.08 g).

We continuously monitored 102 individuals from five captive colonies, each comprising ~20 members on average (colony #1, *n* = 22; colony #2, *n* = 17; colony #3, *n* = 19; colony #4, *n* = 22; and colony #5, *n* = 22), with 24-hour recordings over a 30-day period. Defining one detection event as the moment a single RFID reader senses an individual within its antenna field, we registered a total of 83,693,132 detection events (colony #1: 17,231,837; colony #2: 14,705,185; colony #3: 15,995,068; colony #4: 18,629,270; and colony #5: 17,131,772). From these events, we generated two-dimensional (2D) time-series coordinate data (five by five) at 0.1-s intervals for each individual, yielding ~864,000 data points per individual per day. As shown in Fig. 1C, this setup enabled us to comprehensively track the spatiotemporal locations of all colony members.

Because the RFID-based tracking captured only macroscopic chamber-to-chamber movement rather than fine-scale body movements, we interpreted location information as behavior by linking each chamber to its specific function in the burrow. In the wild, naked mole-rats occupy elongated burrows divided into functionally distinct compartments (*29*). Likewise, under confined laboratory conditions, they partitioned their dwellings into four types of chambers: nest, toilet, garbage, and other (fig. S1A). To investigate the long-term dynamics of these functional chambers, we monitored chamber use twice a day over 18 weeks in three colonies housed in an identical structured system. In these colonies, although typically only one of the nine chambers was designated as each of the three functional types (nest, toilet, and garbage), their stability varied. The toilet and garbage chambers seldom changed location once established (the number of transitions = 42 in total for toilet and *n* = 17 in total for garbage), whereas the nest changed locations frequently (the number of transitions = 217 in total for nest; fig. S1B). This pattern was also evident in the tracking data, where the nest frequently transitioned (*n* = 148 in total), whereas the toilet and garbage rarely transitioned (*n* = 5 in total for toilet and *n* = 2 in total for garbage; Fig. 1D). Based on these findings, for subsequent analyses, the locations of the toilet and garbage chambers were determined manually through multiple daily observations. In contrast, the nest was defined as the chamber with the highest number of individuals staying within a 6-hour window (3 hours before and 3 hours after each observation). Any remaining areas were classified as “other.”

We defined “stay events” as any continuous period during which an individual remained in a single location. Each stay event was categorized according to the function of the chamber (i.e., nest, toilet, garbage, or other). The frequency distribution of event durations revealed that only the nest exhibited two distinct peaks, one 6.3 s and the other 5169.2 s (Fig. 1E). Here, we subdivided nest stay events into two categories, defining those lasting more than 600 consecutive seconds as “Rest.” Consequently, we categorized five types of stay events (Rest, Nest, Toilet, Garbage, and Other) and constructed time-series stay event data for each individual (Fig. 1F).

Because the nine chambers are not spatially equivalent (e.g., corner, edge, or center), we investigated whether spatial positioning might bias chamber usage patterns. Notably, in our system, chamber function was strongly associated with spatial position: Toilet chambers were always located in corners, and garbage chambers tended to be centrally placed (fig. S1C). To isolate the effect of spatial position independent of function, we examined spatial preference patterns focusing on “other” chambers. Across all individuals, stay durations were significantly longer in corner chambers compared to edge or center chambers (fig. S1D), indicating a general preference for remaining in corner positions. No significant differences in stay durations across caste or sex were observed for any chamber position (fig. S1E), suggesting that the spatial preference pattern is determined independently of individual attributes.

When examining the proportion of resting individuals in the colony at each time point, we observed frequent sudden drops in the proportion of resting members to nearly zero (Fig. 1G). Because such drastic declines were indicative of major disturbances to the colony (e.g., incidental noise and vibrations from daily husbandry), we excluded these time points from subsequent analyses. In addition, occasional improbable “leap” movements in the data prevented identification of individual locations in 0.70% of total events (0.43% for #1, 0.15% for #2, 1.30% for #3, 0.49% for #4, and 1.09% for #5; Fig. 1H). After data processing (see Materials and Methods for details), the rate of location errors was reduced to 0.21% (0.06% for #1, 0.07% for #2, 0.34% for #3, 0.18% for #4, and 0.38% for #5; Fig. 1H), which we deemed sufficiently low for subsequent analyses.

### Classification of behavioral phenotypes

**Fig. 2.**
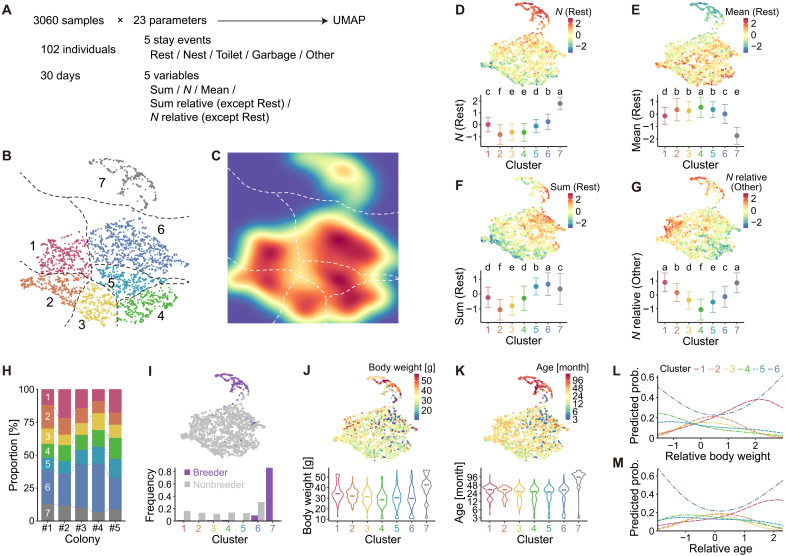
Classification of behavioral phenotypes. (**A** to **C**) Clustering of individual behavioral phenotypes. (A) Schematic of data construction for clustering. (B) UMAP scatter plot of behavioral samples (*n* = 3030), excluding one outlier individual (fig. S2A). Points are colored by cluster assignment based on the segmentation shown in (C). (C) Density map of the UMAP space, segmented into seven compartments using the watershed algorithm. Each sample was assigned to a cluster defined by these compartments. Density is color-coded (red, high; blue, low). (**D** to **G**) Differences among the seven behavioral clusters in four representative parameters: *N* (Rest) (D), Mean (Rest) (E), Sum (Rest) (F), and *N* relative (Other) (G). Upper panels show individual values overlaid on the UMAP scatter plot. Lower panels show means ± SD for each cluster. Different letters indicate significant differences between clusters, based on Holm-adjusted pairwise comparisons of EMMs. Similar comparisons for all 23 parameters are provided in fig. S3. (**H**) Cluster composition across colonies. Stacked bar plots show the proportion of each cluster within each colony. (**I** to **K**) Relationships between cluster assignment and caste (I), body weight (J), or age (K). Upper panels show UMAP scatter plots with points colored by caste (I), body weight (J), or age (K). Lower panels show cluster-wise bar plots grouped by caste (I), and violin plots showing the distribution and medians of body weight (J) or age (K). (**L** and **M**) Relationships between predicted probabilities of cluster assignment and relative body weight (L) or relative age (M) among nonbreeders. Curves represent predictions from multinomial models fitted to nonbreeder samples assigned to clusters 1 to 6 (*n* = 2686).

To classify individual behavioral phenotypes, we used a cluster assignment approach based on dimensionality reduction (*30*). We calculated three behavioral parameters for the five types of stay events (i.e., Rest, Nest, Toilet, Garbage, and Other): total duration (“Sum”), number of events (“*N*”), and mean duration (“Mean”) (Fig. 2A). Because total duration and number of events for each nonresting stay event were expected to scale with an individual’s overall active duration (i.e., the cumulative duration of the four nonresting stay events), we also included “Sum relative” and “*N* relative” for these event types, which were normalized by total active duration. Consequently, our time-series data were transformed into a dataset comprising 3060 samples (102 individuals × 30 days), each represented by a 23-dimensional parameter vector (Fig. 2A). We applied Uniform Manifold Approximation and Projection (UMAP) (*31*) for dimensionality reduction to these samples, generating a 2D scatter plot (Fig. 2B). We then converted the UMAP scatter plot into a density map using Gaussian blurring (Fig. 2C). Applying watershed segmentation to the density map yielded seven total clusters: six gradational clusters (clusters 1 to 6) and one clearly distinct cluster (cluster 7), suggesting distinct behavioral phenotypes.

To determine the characteristics of each cluster, we examined quantitative differences in the behavioral parameters among them (Fig. 2, D to G, and fig. S3). First, when comparing cluster 7 with the others, we found that it had a significantly higher number of rest events [*N* (Rest)] and a significantly lower mean duration of rest [Mean (Rest)] than any other cluster (Fig. 2, D and E). In contrast, the total duration of rest [Sum (Rest)] did not differ notably between cluster 7 and the other clusters (Fig. 2F). These findings suggest that cluster 7 exhibits a distinctive resting pattern characterized by frequent, short rest events. Another notable feature of cluster 7 is its high value for *N* relative (Other) (Fig. 2G), one of the two highest across all clusters. Because the number of “Other” events was strongly and positively correlated with how often individuals moved between adjacent areas (Pearson correlation test, *r* = 0.991, *t*_3058_ = 412.11, *P* < 0.001), *N* relative (Other) reflects overall movement tendencies during active periods. Furthermore, during these active periods, individuals in cluster 7 spent more time in the nest and less time in other chambers than did those in the remaining clusters (fig. S3, B to E). Together, these observations indicate that individuals in cluster 7 display three key behavioral characteristics: (i) a unique resting pattern with frequent but brief rests, (ii) a high propensity for movement, and (iii) prolonged nest occupancy during active periods.

Next, we examined quantitative differences in behavioral parameters among the six gradational clusters (clusters 1 to 6). The UMAP 2D scatter plot revealed clear distinctions across these clusters, particularly regarding (i) overall activity duration, as inversely represented by Sum (Rest), and (ii) movement tendency, as indicated by *N* relative (Other). Consequently, individuals in clusters 2 and 3 were relatively hyperactive [i.e., low Sum (Rest)], whereas those in clusters 5 and 6 were hypoactive [i.e., high Sum (Rest)] (Fig. 2F). Furthermore, because *N* relative (Other) reflects movement tendencies, cluster 1 exhibited a high level of movement, whereas cluster 4 showed a low level of movement (Fig. 2G). When accounting for activity duration, individuals in cluster 5 showed the highest occupancy in toilet [Sum relative (Toilet)], whereas those in cluster 4 exhibited the lowest (fig. S3C). Individuals in clusters 1 and 2 spent more time in garbage [i.e., high Sum relative (Garbage)] (fig. S3D), a pattern partially associated with their high mobility. In contrast, Sum relative (Other) was inversely related to mobility: Individuals in cluster 4 showed the highest occupancy, whereas those in cluster 1 showed the lowest (fig. S3E). These distinct behavioral patterns across clusters may reflect differentiated functional roles within the colony.

We examined the relationships between the clusters and individual attributes. The cluster composition for each colony is presented in Fig. 2H, and no substantial bias was found across colonies. There was a significant difference in cluster assignment between breeders and nonbreeders [likelihood ratio test (LRT), χ^2^ = 1309.50, df = 6, *P* < 0.001]. Breeders were disproportionately assigned to cluster 7 (86.3%), whereas nonbreeders were distributed across clusters other than cluster 7 (Fig. 2I), which indicates a clear difference in behavioral phenotypes between breeders and nonbreeders. Body weight and age of individuals had significant effects on cluster assignment [body weight: LRT, χ^2^ = 524.55, df = 6, *P* < 0.001 (Fig. 2J); age: LRT, χ^2^ = 1090.60, df = 6, *P* < 0.001 (Fig. 2K)], which aligns with the larger body size and older age of breeders (cluster 7).

To explore the factors influencing cluster assignment in nonbreeders, we examined the effect of relative body weight or relative age within the colony on cluster assignment. Then, we found significant effects of relative body weight (LRT, χ^2^ = 394.22, df = 10, *P* < 0.001) and relative age (LRT, χ^2^ = 273.78, df = 10, *P* < 0.001). Specifically, the predicted probabilities for assignment to cluster 6 exhibited a U-shaped distribution in relative body weight and relative age (Fig. 2, L and M), suggesting that nonbreeders who were not intermediate in body size or age were more likely to be assigned to cluster 6. On the other hand, the predicted probabilities for assignment to clusters 1 to 5 exhibited unimodal distributions with peaks that differed consecutively (Fig. 2, L and M). In addition, sex had a significant effect on cluster assignment in nonbreeders (LRT, χ^2^ = 30.65, df = 5, *P* < 0.001; fig. S2B). When examining the effects of relative body weight or relative age accounting for sex, significant interactions were found between sex and relative body weight (LRT, χ^2^ = 394.22, df = 10, *P* < 0.001) or sex and relative age (LRT, χ^2^ = 273.78, df = 10, *P* < 0.001) on cluster assignment. In particular, larger/older nonbreeding males, rather than nonbreeding females, were markedly more likely to be assigned to cluster 1 (fig. S2, C and D). These findings suggest that differences in individual attributes have a notable, although not decisive, influence on the separation of nonbreeders’ behavioral phenotypes.

### Consistency of behavioral phenotypes

**Fig. 3.**
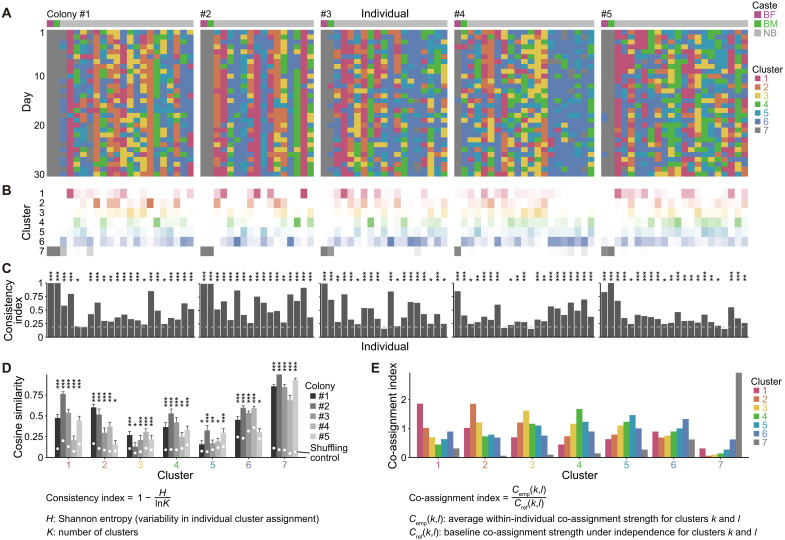
Consistency of behavioral phenotypes. (**A**) Behavioral cluster assignments for each individual on each of the 30 days. (**B**) Summary of cluster assignment frequency per individual across the 30 days; darker color indicates higher frequency. (**C**) Consistency index for each individual across the days (*n* = 101 individuals). The calculation formula is shown below the figure. Dashed lines represent the 95% upper bound of the simulated values. Statistical significance was assessed by comparing each observed value to the simulated distribution. (**D**) Cosine similarity between consecutive days for each cluster in each colony (*n* = 29 per cluster per colony). Bars show means ± SE for visualization. White circles represent the shuffling control (mean of shuffled values). Statistical comparisons (observed versus shuffled) used the Wilcoxon rank-sum test. (**E**) Co-assignment index for each cluster pair (square root–transformed for visualization). The calculation formula is shown below the figure. *P < 0.05; **P < 0.01; ***P < 0.001.

To assess the stability of the identified clusters, we investigated the extent to which each individual remained in the same cluster over 30 days (Fig. 3, A and B). For each individual, we calculated a “consistency index” that quantifies how consistently individuals were assigned to a single cluster (Fig. 3C). Although consistency index varied widely among individuals, the vast majority (96 of 101 individuals, 95.0%) showed significant consistency (Fig. 3C). These findings suggest that most naked mole-rats maintain a stable behavioral phenotype over time. Next, to determine how stable each behavioral cluster remained within each colony, we computed cosine similarities for each cluster across two consecutive days. In all clusters of all colonies, these cosine similarities were significantly higher than expected by randomized data (Fig. 3D), indicating robust stability at the colony level. Last, to examine how different behavioral clusters were assigned within individuals across days, we calculated a “co-assignment index” for all possible cluster pairs. We found that the highest co-assignment index was for identical cluster pairings (Fig. 3E). For different cluster pairings, those that were closer on the UMAP 2D scatter plot (Fig. 2B) tended to show higher co-assignment indices (Fig. 3E). These results imply that individual naked mole-rats exhibit consistent behavioral phenotypes, while occasionally undergoing low-frequency transitions among similar behavioral phenotypes.

In addition to examining consistent behavioral phenotypes, we investigated whether individuals differed in their roles during collective nest transitions, a key event in group coordination. We quantified the order in which individuals began resting in the newly selected nest for each transition (fig. S4A). Although no single prominent leader emerged, some individuals consistently initiated transitions earlier than others (fig. S4B). Leadership tendency was highest in cluster 7 (breeders); among nonbreeders, individuals in clusters 5 and 6 exhibited higher leadership tendencies, whereas those in clusters 2 and 3 showed lower tendencies (fig. S4C). These results suggest that nest transitions are not entirely stochastic but are influenced by breeders’ contributions. Among nonbreeders, variation in leadership tendencies largely mirrored rest frequency (Fig. 2F), suggesting that early rest initiation may reflect general activity levels rather than specific leadership roles.

### Synchronization of activity rhythms

**Fig. 4.**
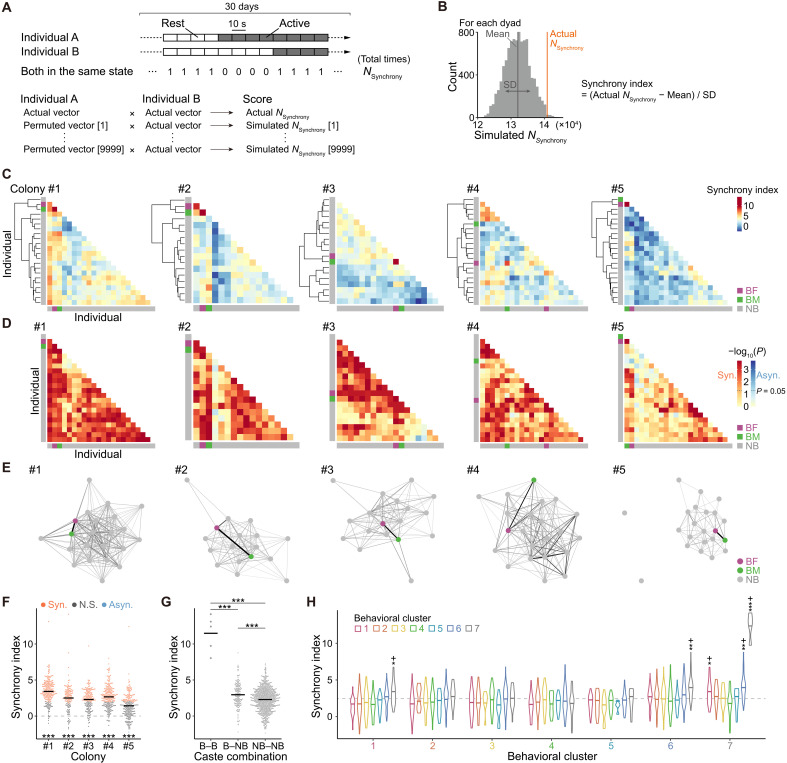
Synchronization of activity rhythm. (**A** and **B**) Calculation flow for activity rhythm synchrony indices. (A) *N*_Synchrony_ was calculated, and 9999 simulated datasets were generated per dyad. (B) Synchrony index was derived from the position of the actual *N*_Synchrony_ within the simulated distribution (see formula in the figure). (**C**) Heatmap of synchrony indices by dyad for each colony with hierarchical clustering. Axes represent individuals. (**D**) Heatmap of *P* values by dyad for each colony. Axis order matches panel (C). Red/blue scales indicate whether the actual *N*_Synchrony_ is above or below the simulated median, respectively. Dark red and dark blue (*P* < 0.05) indicate significantly synchronous (Syn.) or asynchronous (Asyn.) dyads, respectively. (**E**) Undirected, weighted network of significantly synchronous dyads. Nodes, individuals; edges, synchrony indices (darker/thicker = higher). (**F**) Synchrony indices by colony, compared to the shuffling control (dashed line) using one-sample *t* tests on EMMs. N.S., nonsignificant. Bars indicate estimates from an LM. *n*: #1 = 231; #2 = 136; #3 = 171; #4 = 231; and #5 = 231. (**G**) Comparison of synchrony indices across caste combinations. Bars indicate estimates from an LMM. *n*: B-B = 5; B-NB = 184; and NB-NB = 811. Asterisks indicate significant differences based on Holm-adjusted pairwise comparisons of EMMs. (**H**) Synchrony indices for each behavioral cluster combination, compared to the observed mean (dashed line) using one-sample *t* tests on EMMs. Bars indicate estimates from an LMM (*n* = 979). See table S1 for sample sizes per combination. *P < 0.05; **P < 0.01; ***P < 0.001. B, breeder; NB, nonbreeder.

Various social animals synchronize their activity rhythms with group members through a combination of innate circadian cycles and social synchronization effects (*32*–*37*). However, naked mole-rats typically lack a circadian rhythm while residing in their colony (*38*, *39*), implying a heterogeneous degree of rhythm synchronization within the colony. Thus, to investigate whether social synchronization drives activity rhythm synchrony, we measured it for every pair (dyad) of individuals. Specifically, at 10-s intervals over a 30-day recording, we counted how often two individuals were in the same activity state, either both resting (“Rest”; Fig. 1E) or both active (“Nest,” “Toilet,” “Garbage,” and “Other”; Fig. 1E). We refer to its total counts as *N*_Synchrony_ (Fig. 4A). To determine how significantly the activity rhythm synchrony of each dyad differed from chance, we performed a permutation test by comparing the actual data with shuffled datasets. For every 10-s interval over 30 days, we created a binary time-series vector of “Rest” or “Active” for each individual. To generate a null distribution, we created 9999 randomly permuted vectors for one member of each dyad by cyclically fixing the sequence and then counted *N*_Synchrony_ in the same way for each permutation. By comparing the actual data of *N*_Synchrony_ with the 9999 simulated values, we calculated a “synchrony index” and a *P* value for each dyad (Fig. 4B). Consequently, we visualized synchrony indices and *P* values of all dyads in matrix form (Fig. 4, C and D) and extracted dyads that were significantly synchronous to construct a network for each colony (Fig. 4E).

We explored the relationship between activity rhythm synchrony and attribute factors such as caste and sex. Overall, synchrony indices were significantly greater than the shuffling control in all colonies (Fig. 4F). A majority of dyads (62.6 to 81.8%) were significantly synchronous in colonies #1 to 4 (Fig. 4F, red dots, and fig. S5A), whereas only two dyads, one in colony #3 and one in colony #5, were significantly asynchronous (Fig. 4F, blue dots, and fig. S5A). These findings indicate that naked mole-rats often synchronize their activity rhythms with colony members; however, not all individuals synchronize with each other, and some dyads exhibit independent activity rhythms. Furthermore, in comparing synchrony indices across breeders and nonbreeders, breeder-breeder dyads showed markedly higher values (Fig. 4G), indicating that breeders strongly align their activity rhythms with each other. Breeder-nonbreeder dyads also had significantly higher synchrony indices than nonbreeder-nonbreeder dyads (Fig. 4G), suggesting that breeders positively influence activity rhythm synchrony. In breeder-nonbreeder dyads, we found no significant differences in synchrony indices among any sex combinations (fig. S5B), whereas the female-female combinations had significantly higher synchrony indices than the other combinations in nonbreeder-nonbreeder dyads (fig. S5C). These results indicate that sex has a partial but minor effect on activity rhythm synchrony.

To examine the relationship between activity rhythm synchrony and behavioral phenotypes, we analyzed synchrony indices for dyads comprising each possible combination of the seven behavioral clusters (Fig. 4H). Dyads in which both individuals belonged to cluster 7 exhibited the highest synchrony indices, consistent with the markedly high values observed among breeder-breeder dyads (Fig. 4G). Moreover, cluster 1–cluster 7 dyads and cluster 6–cluster 7 dyads showed high synchrony indices (Fig. 4H), which also aligns with the elevated synchrony observed in breeder-nonbreeder dyads (Fig. 4G). These findings suggest that breeders have a partially positive influence on the activity rhythm synchrony of nonbreeders exhibiting specific behavioral phenotypes.

To evaluate the temporal stability of individual roles, we calculated strength centrality for each individual in six nonoverlapping 5-day subsets. Strength centralities in the synchrony network showed clear and consistent individual differences across the 5-day subsets (fig. S5E), suggesting that certain individuals consistently played central roles within the synchrony network. Notably, individuals in cluster 7 consistently displayed high strength centrality in the synchrony network, whereas those in cluster 4 showed lower values (fig. S5F). These results indicate that breeders consistently serve as central figures within the synchrony network.

### Spatial proximity

**Fig. 5.**
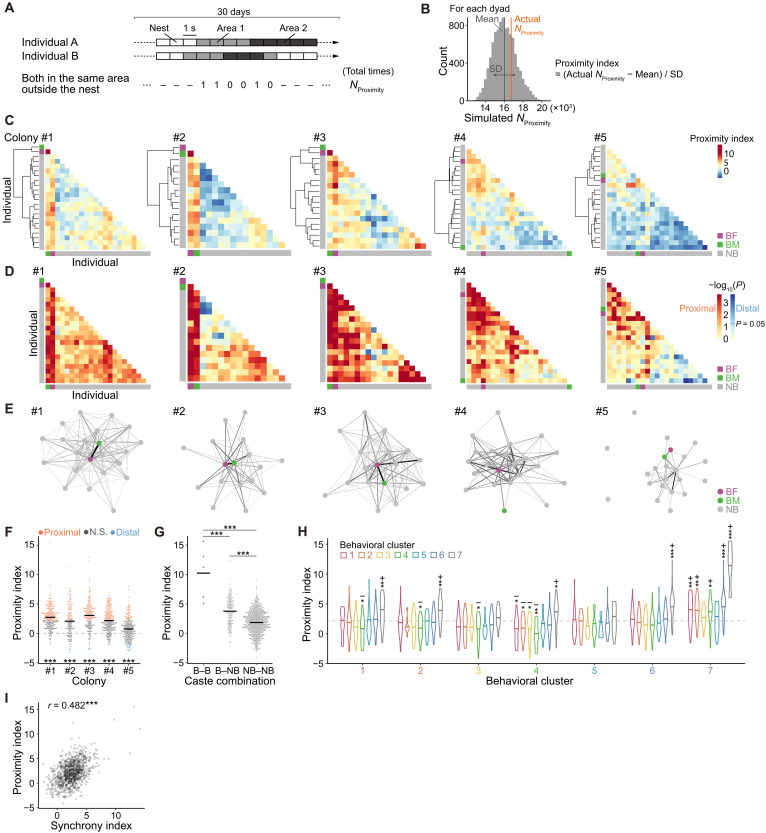
Spatial proximity. (**A** and **B**) Calculation flow for spatial proximity indices. (A) *N*_Proximity_ was calculated, and 9999 simulated datasets were generated per dyad. (B) Proximity index was derived from the position of the actual *N*_Proximity_ within the simulated distribution (see formula in the figure). (**C**) Heatmap of proximity indices by dyad for each colony with hierarchical clustering. Axes represent individuals. (**D**) Heatmap of *P* values by dyad for each colony. Axis order matches panel (C). Red/blue scales indicate whether the actual *N*_Proximity_ is above or below the simulated median, respectively. Dark red and dark blue (*P* < 0.05) indicate significantly proximal or distal dyads, respectively. (**E**) Undirected, weighted network of significantly proximal dyads. Nodes, individuals; edges, proximity indices (darker/thicker = higher). (**F**) Proximity indices by colony, compared to the shuffling control (dashed line) using one-sample *t* tests on EMMs. Proximal, significantly proximal; distal, significantly distal. Bars indicate estimates from an LM. *n*: #1 = 231; #2 = 136; #3 = 171; #4 = 231; and #5 = 231. (**G**) Comparison of proximity indices across caste combinations. Bars indicate estimates from an LMM. *n*: B-B = 5; B-NB = 184; and NB-NB = 811. Asterisks indicate significant differences based on Holm-adjusted pairwise comparisons of EMMs. (**H**) Proximity indices for each behavioral cluster combination, compared to the observed mean (dashed line) using one-sample *t* tests on EMMs. Bars indicate estimates from an LMM (*n* = 979). See table S1 for sample sizes per combination. (**I**) Relationship between synchrony and proximity indices. Pearson’s correlation coefficient (*r*) is shown. *P < 0.05; **P < 0.01; ***P < 0.001. B, breeder; NB, nonbreeder.

It is common to assess social connections within groups by measuring spatial proximity rather than direct social interactions (*40*–*43*). Naked mole-rats typically remain highly aggregated in their nest, habitually huddling together during resting periods (*44*). However, during active periods, they engage in various behaviors and disperse throughout the burrow. Consequently, spatial proximity can vary substantially among dyads within a colony. Thus, following a procedure similar to our analysis of activity rhythm synchrony (Fig. 4), we quantified spatial proximity by counting, at 1-s intervals over a 30-day recording, how often two individuals were (i) both in an active state, (ii) simultaneously outside the nest, and (iii) in the same arena (*N*_Proximity_; Fig. 5A). We then compared these observed *N*_Proximity_ values with 9999 randomly permuted datasets to derive a “proximity index” and *P* value for each dyad (Fig. 5B). Thus, we visualized the proximity indices and *P* values for all dyads (Fig. 5, C and D) and constructed a network of significantly proximal dyads for each colony (Fig. 5E). Overall, proximity indices were significantly higher than chance in all colonies (Fig. 5F). Nearly half of the dyads (45.6 to 66.7%) were significantly proximal in colonies #1 to 4 (Fig. 5F, red dots, and fig. S5A), whereas few dyads (0.0 to 8.2%) were significantly distal (Fig. 5F, blue dots, and fig. S5A). These results suggest that naked mole-rats commonly engage in activities near specific colony members, yet a notable fraction of dyads exhibit a lack of distinct spatial association. Moreover, breeder-breeder dyads again showed the highest proximity indices (Fig. 5G), suggesting that breeders exert a strong mutual influence on their spatial cohesion. Breeder-nonbreeder dyads were also more proximal than nonbreeder-nonbreeder dyads (Fig. 5G), mirroring the pattern observed in activity rhythm synchrony. Notably, within breeder-nonbreeder dyads, breeding females had significantly higher proximity indices than breeding males (fig. S6B). No substantial sex-related differences emerged in nonbreeder-nonbreeder dyads (fig. S6C). These results suggest the strength of the influence breeding females have on spatial proximity.

Next, to clarify how behavioral phenotypes affect proximity, we analyzed the proximity indices for dyads comprising every possible combination of the seven behavioral clusters (Fig. 5H). Dyads in which both individuals belonged to cluster 7 exhibited the highest proximity indices, consistent with the markedly high values observed among breeder-breeder dyads (Fig. 5G). Several other combinations involving cluster 7, specifically cluster 1–cluster 7, cluster 2–cluster 7, cluster 4–cluster 7, and cluster 6–cluster 7, also showed elevated proximity (Fig. 5H), correlating with the high proximity in breeder-nonbreeder dyads (Fig. 5G), which suggests that breeders can positively influence certain nonbreeders’ spatial choices. In contrast, several combinations involving cluster 4, specifically cluster 1–cluster 4, cluster 2–cluster 4, cluster 3–cluster 4, and cluster 4–cluster 4, had significantly lower proximity indices (Fig. 5H), suggesting that individuals belonging to cluster 4 tend to avoid other active workers (clusters 1 to 4). Overall, these findings highlight a partial yet consistent role of breeders in maintaining spatial proximity, while revealing that certain nonbreeder phenotypes (e.g., cluster 4) participate less frequently in shared activity spaces.

Using the same time-split approach as the synchrony network (fig. S5, E and F), we evaluated strength centrality in the proximity network. Strength centralities in the proximity network also exhibited stable individual differences across subsets (fig. S6E). Individuals in cluster 7 consistently exhibited markedly high strength centrality in the proximity network, whereas those in clusters 3 and 4 showed lower values (fig. S6F), indicating that breeders also serve as central figures in the spatial association networks.

At the colony-wide level, spatial proximity and activity rhythm synchrony were positively correlated across dyads (Fig. 5I; positive correlations were consistent across colonies). Nevertheless, a more fine-grained analysis based on each behavioral phenotype captured the deeper social complexity within naked mole-rat colonies.

### Directional following relationships

**Fig. 6.**
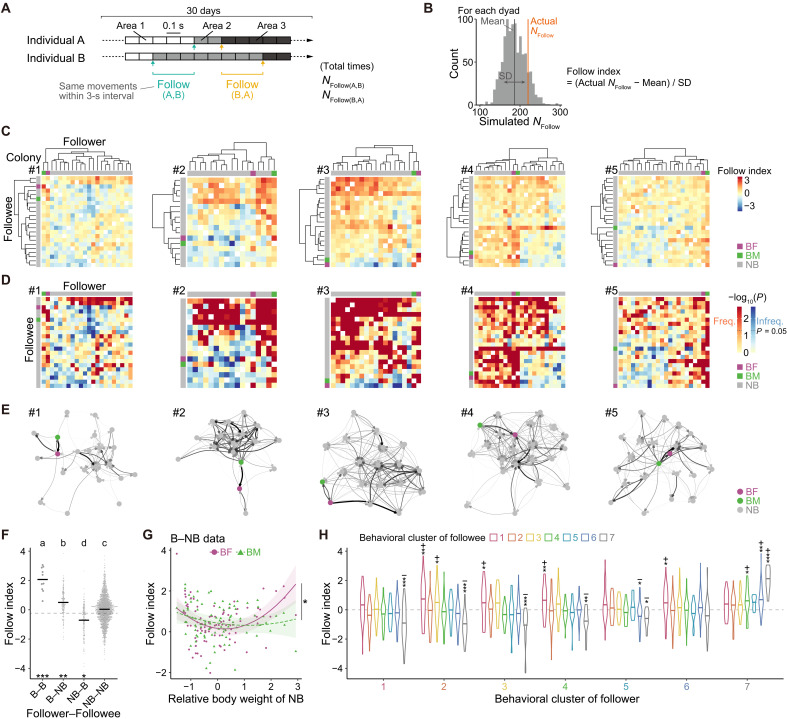
Directional following relationships. (**A** and **B**) Calculation flow for follow indices. (A) *N*_Follow_ was calculated, and 999 simulated datasets were generated per directed dyad. (B) Follow index was derived from the position of the actual *N*_Follow_ within the simulated distribution (see formula in the figure). (**C**) Heatmap of follow indices by directed dyad for each colony with hierarchical clustering. *x* axis, follower ID; *y* axis, followee ID. (**D**) Heatmap of *P* values by directed dyad for each colony. Axis order matches panel (C). Red/blue scales indicate whether the actual *N*_Follow_ is above or below the simulated median, respectively. Dark red and dark blue (*P* < 0.05) indicate directed dyads that exhibit significantly more (Freq.) or less (Infreq.) frequent following, respectively. (**E**) Directed, weighted network of directed dyads that showed significantly more frequent following. Nodes, individuals; edges, follow indices (darker/thicker = higher). Arrows are directed from the follower to the followee. (**F**) Comparison of follow indices across caste pairs (follower-followee). Bars indicate LMM estimates. *n*: B-B = 10; B-NB = 184; NB-B = 184; and NB-NB = 1622. Different letters indicate significant differences based on Holm-adjusted pairwise comparisons of EMMs. Asterisks indicate significant differences from the shuffling control (dashed line). (**G**) Relationship between follow indices and nonbreeder’s relative body weight across breeder sex (B-NB dyads). Curves, LMM predictions (*n* = 92/group); asterisk, a significant interaction between breeder sex and nonbreeder’s relative body weight. (**H**) Follow indices for each behavioral cluster pair, compared to the observed mean (dashed line) using one-sample *t* tests on EMMs. Bars indicate LMM estimates (*n* = 1958). See table S1 for sample sizes per pair. *P < 0.05; **P < 0.01; ***P < 0.001. B, breeder; NB, nonbreeder.

Social relationships can also be investigated by examining directional interactions (*15*, *45*–*48*). In naked mole-rat colonies, individuals often follow other members in various contexts, and the frequency of such follow behavior can vary substantially depending on their relationship. Here, we characterized directional relationships by analyzing the frequency of follow behavior for each directed dyad. Thus, we quantified follow behavior by counting how often, within 3 s after one individual (the followee) in each directed dyad moved between adjacent areas, the other individual (the follower) moved along the same path (*N*_Follow_; Fig. 6A). To determine whether each directed dyad’s follow frequency significantly differed from chance, we performed a permutation test by comparing the actual data with shuffled datasets, as in Figs. 4 and 5. Over the 30-day recording, we generated time-series vectors of movement events (timing and path) for each individual. To create a null distribution, we produced 999 randomly permuted path vectors for one member of each dyad by cyclically fixing the sequence and then counted *N*_Follow_ in the same manner for each permutation. Comparing the observed *N*_Follow_ with the simulated values yielded a “follow index” and *P* value for each directed dyad (Fig. 6B). We then visualized the follow indices and *P* values for all directed dyads in matrix form (Fig. 6, C and D) and constructed a network of directed dyads showing significantly frequent follow behavior for each colony (Fig. 6E).

We next investigated how directional following varied with key attributes. Although significantly more frequent following (14.5 to 48.8%; fig. S7A) occurred more often than significantly less frequent following (4.5 to 13.4%; fig. S7A), nonsignificant associations remained the most prevalent across all colonies (45.6 to 72.1%; fig. S7A). These results suggest dyad variation in follow behavior, with most dyads showing no distinct following relationships. Breeder-breeder dyads had significantly higher follow indices than both the shuffling control and all other caste pairs (Fig. 6F), indicating that breeders follow each other at a high frequency. More notably, breeders followed nonbreeders significantly more often than expected by chance, whereas nonbreeders rarely reciprocated—following breeders significantly less often than chance (Fig. 6F). This asymmetry contrasts with the more balanced patterns observed for activity rhythm synchrony and spatial proximity. In nonbreeder-nonbreeder dyads, follow indices did not differ from chance, suggesting that nonbreeders follow one another largely at random. We then explored whether sex influenced these relationships. Breeding males tended to follow breeding females more frequently than the reverse, although this trend did not reach statistical significance (paired *t* test, *t*_4_ = −2.30, *P* = 0.083; fig. S7B). Within breeder-nonbreeder and nonbreeder-breeder dyads, follow indices did not differ significantly by sex (fig. S7, C and D). Among nonbreeders, however, nonbreeding males were followed significantly more often than nonbreeding females (fig. S7E). Notably, breeding females exhibited a marked preference for following larger nonbreeders, an effect not observed in breeding males (LRT, χ^2^ = 8.23, df = 2, *P* = 0.016; Fig. 6G), whereas relative age produced no comparable interaction (LRT, χ^2^ = 4.58, df = 2, *P* = 0.101; fig. S7F).

To complement the tracking data and better characterize the social context of follow events, we conducted a video-based analysis of interaction types. For each of the four breeder/nonbreeder dyad classes, we randomly selected 100 follow events and reviewed the corresponding video footage (breeder-breeder: 9182; breeder-nonbreeder: 41,264; nonbreeder-breeder: 28,345; and nonbreeder-nonbreeder: 223,976 events in total) (fig. S8A). Each video segment was manually annotated into one of four interaction categories (fig. S8B): affiliative (e.g., mating, anogenital nuzzling, and play fighting; movies S1 to S3), agonistic (e.g., shoving and biting; movies S4 and S5), tail tugging (a behavior in which one individual pulls the tail of another, typically with the tail-puller recorded as the followee; movie S6) (*49*), and neutral (no discernible interaction). Neutral interactions were the most common across all dyad classes (fig. S8C). However, affiliative interactions were observed in 46% of breeder-breeder events, whereas 32% of breeder-nonbreeder events involved agonistic behaviors (fig. S8C). In contrast, nonbreeder-breeder and nonbreeder-nonbreeder follow events were rarely affiliative or agonistic, although tail tugging occurred relatively frequently in nonbreeder-nonbreeder dyads (fig. S8C). These findings clarify the behavioral content underlying follow events, revealing distinct interaction patterns across dyad classes and underscoring the context-dependent nature of follow behavior.

To clarify how behavioral phenotypes shape these directional following patterns, we assessed follow indices for every possible directed pair of the seven behavioral clusters (Fig. 6H). Breeder-breeder pairs (cluster 7–cluster 7) again stood out with the highest follow indices. Consistent with the asymmetry detected in breeder-nonbreeder following (Fig. 6F), breeders (cluster 7) frequently followed nonbreeders from clusters 4 and 6, whereas nonbreeders from clusters 1 to 5 rarely followed breeders (cluster 7) (Fig. 6H). In addition, cluster 1 (typically high-mobility workers; Fig. 2, D to G) attracted frequent following from multiple clusters (clusters 2, 3, 4, and 6). Collectively, these findings emphasize that following behavior is not merely a reflection of overall synchrony or proximity but rather reveals distinct, directional patterns of social interaction, driven by specific caste- and behavioral phenotype–related differences.

Using the same time-split approach as in the synchrony and proximity network (figs. S5, E and F, and S6, E and F), we calculated out-strength and in-strength centrality for each individual across six 5-day subsets. Both measures demonstrated stable individual differences (fig. S7, H and I). Individuals in cluster 7 frequently initiated following (high out-strength) but were infrequently followed by others (low in-strength), whereas those in cluster 1 showed the opposite pattern (fig. S7, J and K). These findings indicate that breeders play central roles in the directional following network as well.

## DISCUSSION

In this study, we used an RFID-based tracking system to monitor entire naked mole-rat colonies under steady-state conditions, providing long-term, comprehensive behavioral profiling. Although image-based methods such as barcode tracking (*10*, *11*) and markerless tracking with deep learning (*12*, *13*) offer high-resolution analyses, RFID has two key advantages for multianimal studies. First, it greatly minimizes identification errors because each individual carries an embedded microchip, ensuring detection even when animals overlap in narrow tunnels. By contrast, image-based tracking can fail when multiple individuals cluster together, increasing invisibility and mismatches (*11*, *50*, *51*). Second, compared to image-based methods, RFID tracking generates simpler, easily processed data and requires far fewer computing resources, making it well suited for continuous, long-term observation (*15*, *16*, *52*). Furthermore, the RFID readers used here detect multiple individuals simultaneously, maintaining high accuracy as colony size grows. Overall, this system addresses the unique challenges of naked mole-rat behavior and offers a powerful platform for investigating their complex social dynamics.

Our study quantitatively revealed three key features of naked mole-rat breeders: (i) frequent short rests, (ii) strong social bonds within breeding pairs, and (iii) subtle sex differences in breeder behavior. Breeders uniquely exhibit frequent short rests (Fig. 2, D, E, and I), possibly reflecting repeated patrols and dominant interactions. Breeding females patrol burrows and display agonistic dominance toward nonbreeders (fig. S8, B and C) (*27*, *53*–*55*), paralleling queen aggression in primitively eusocial insects (*56*–*59*). Because dominant breeder behavior involves a form of following, namely, displacement through shoving (fig. S8B) (*53*, *60*), our observation of breeders following nonbreeders (Fig. 6F) supports this interpretation. Moreover, breeding pairs form exceptionally strong bonds: They show highly synchronous activity rhythms, remain close even while active, and frequently follow each other (Figs. 4G, 5G, and 6F), consistent with an earlier observational study (*61*) describing their frequent sexual interactions and close nest occupancy. Although breeding females and males do not differ in overall synchrony or spatial proximity with nonbreeders (figs. S5B and S6B), breeding females move ahead of breeding males more often (fig. S7B) and follow larger nonbreeders more frequently (Fig. 6G). This pattern aligns with their pronounced intracolony aggression toward higher-ranking nonbreeders (*27*, *53*), indicating that breeding females occupy a more proactive role in the social hierarchy.

Our findings highlight the behavioral heterogeneity of nonbreeding naked mole-rats, providing further insights into “polyethism” within their colonies. Previous studies have suggested that nonbreeders’ behavioral phenotypes are continuous rather than discrete (*20*, *25*, *26*), consistent with our classification (Fig. 2, B and C). In addition to overall activity and mobility, behavioral clusters differed in their patterns of engagement with functionally distinct chambers, such as nest, toilet, and garbage chambers. These differences may reflect a form of task allocation: For example, cluster 1 individuals (high mobility and garbage occupancy) may serve as transport specialists, whereas those in cluster 4 (low mobility and frequent occupancy of nonfunctional chambers) may engage primarily in digging tasks. Cluster 5 individuals, who frequently occupied toilet chambers, may contribute to cleaning-related role. These functional differences among clusters suggest that differentiated behavioral roles may emerge in association with broader life-history trait. The proximity of clusters 6 and 7 in behavioral space (Fig. 2B), particularly in terms of rest-related parameters and nest occupancy (fig. S3, A and B), suggests that both clusters represent low-activity phenotypes centered around the nest. This behavioral overlap may account for the relatively fluid transitions observed between these clusters. Notably, variations in these behavioral clusters are closely tied to individual age and body size, factors previously linked to polyethism in cooperatively breeding vertebrates (*20*–*26*, *62*–*64*), and may explain why nearly all individuals showed stable behavior over 30 days (Fig. 3C).

Moreover, behavioral phenotypes correlate with social relationships (Figs. 4H, 5H, and 6H). For instance, low-mobility nonbreeders (cluster 4) tended to avoid other active workers (Fig. 5H). This pattern enables nonbreeders to regulate spatial proximity for various tasks: Although co-working in the same place [e.g., digging chains; (*65*)] can enhance efficiency (*66*, *67*), working independently may be more effective for exploratory foraging (*68*, *69*). We observed comparable numbers of dyads that stayed proximal or were fairly independent, with relatively few dyads that actively avoided each other (Fig. 5F and fig. S5A). In addition, high-mobility nonbreeders (cluster 1) attracted frequent following (Fig. 6H and fig. S7K). Among nonbreeders, follow events frequently involve tail tugging (fig. S8C), where the tail-puller typically becomes the followee (*49*). Given this, the frequent followee status of high-mobility nonbreeders suggests that they may commonly initiate tail-tugging interactions.

Behavioral clusters may be differentially associated with defensive roles. Previous studies suggest that older and heavier nonbreeders are more likely to engage in defense (*20*–*22*). Although our design did not include intruder tests, clusters 1 and 6, both containing older and heavier individuals, may reflect distinct defensive strategies. Cluster 1’s high mobility may support a patrol-like role, as seen in ants where exploratory individuals also engage in defense (*70*, *71*). In contrast, cluster 6’s low activity may parallel the reactive, standby role of termite soldiers (*72*).

Despite providing large-scale, continuous behavioral data, our RFID-based system did not capture fine-grained movement or posture within each chamber, preventing us from confirming specific behaviors (e.g., digging) or direct interactions among co-occupants. Incorporating image-based methods (*10*–*13*, *51*) or focused experimental tests could resolve these details, as demonstrated in mouse studies combining both approaches (*15*, *16*). Moreover, although our colonies (~20 individuals) reflect a typical size in the wild (*73*), larger colonies may feature more distinct behavioral structures or increased task specialization (*74*, *75*). Future research examining a wider range of colony sizes and ages will thus clarify how behavioral phenotypes and social relationships vary in naked mole-rats.

Overall, our findings reveal the distinct roles of breeders and the remarkable behavioral diversity among nonbreeders, underscoring the complexity of naked mole-rat social organization. By adopting a scalable RFID-based system for long-term, multianimal tracking, we have demonstrated how large-scale data collection can illuminate both individual behaviors and collective dynamics. Future studies that integrate experimental manipulations or additional observation methods promise to deepen our understanding of eusociality in this unique mammalian system and uncover underlying mechanisms driving cooperative society.

## MATERIALS AND METHODS

### Animals

We used naked mole-rats (*n* = 102; *n* = 64 females, *n* = 38 males) from five captive colonies maintained at the Department of Aging and Longevity, Faculty of Life Sciences, Kumamoto University, Japan. The colony compositions were as follows: colony #1: *n* = 22 (14 females, 8 males), colony #2: *n* = 17 (13 females, 4 males), colony #3: *n* = 19 (12 females, 7 males), colony #4: *n* = 22 (11 females, 11 males), and colony #5: *n* = 22 (14 females, 8 males). Each colony included one breeding female and one breeding male, identified based on sexual interactions and dominant behavior. All individuals were marked with tattoos for identification, and information on caste, sex, and age was recorded. The animals were housed in artificial burrow systems composed of clear acrylic boxes connected by pipes. Burrow size varied depending on group size, with total length ranging from 1.4 to 2.0 m. Tissue paper was provided as nesting material every other day. Housing conditions were maintained at 30 ± 0.5°C and 60 ± 5% humidity under a 12/12-hour light/dark cycle. The animals were fed sweet potatoes, carrots, and apples three times a week; bananas and oatmeal twice a week; and a small amount of mouse pellets as a supplement once a week. The procedure used in this study was approved by the Ethics Committee of Kumamoto University (approval no. A2020-042), whose guidelines are in accordance with the Guide for the Care and Use of Laboratory Animals (United States National Institutes of Health, USA).

### Animal tagging

All 102 animals were subcutaneously implanted with a single RFID microchip (2.1 mm in diameter by 12 mm in length, 0.08 g; Phenovance LLC, Japan) under isoflurane anesthesia. Body weight was recorded just before implantation. After implantation, each individual was placed in a separate cage to recover from anesthesia. Once recovered, the individual was then introduced into the experimental housing system immediately after recovery. This procedure did not result in any mortality or disease, and no observable effects on behavior were noted.

### Behavioral setup

A colony of naked mole-rats was introduced into the experimental housing system at least 3 days before the start of behavioral recording. The housing system consisted of nine acrylic boxes (15 cm by 15 cm by 20 cm) arranged in a three-by-three grid on a flat surface, with adjacent boxes connected by acrylic pipes (5-cm inner diameter by 16-cm long). A total of 24 RFID readers (Phenovance LLC, Japan) with ring-shaped antennas (6-cm inner diameter) were attached to both ends of each pipe. The records from this RFID system were collected continuously for 24 hours per day over 30 days within a 2-month period for each colony. Throughout the entire recording period, the animals were allowed to move freely within the housing system. The animals were fed once daily during the daytime (11:00 to 19:00), and additional tissue paper was provided, with soiled paper being replaced as needed. The housing system was continuously videotaped with an infrared-compatible camera (ATOM Cam 2, ATOM Tech Inc., Japan) positioned directly above.

### Animal tracking

The RFID system recorded the time, individual identity, and reader location. The RFID readers used in this study were capable of detecting multiple individuals simultaneously, enabling comprehensive detection of crowded movements. The detection event data were structured into records at 0.1-s intervals. The housing system was modeled as a five-by-five 2D grid, with each box or pipe occupying one square. Based on the detection event data, each individual was mapped onto the corresponding position in the five-by-five grid at each time point. Specifically, an individual detected by a reader was defined as being located within the pipe where the reader was installed at the time of detection. When an individual was detected by one reader and subsequently by another, it was assumed to have remained between the two readers during the interval. When an individual was detected by a reader and subsequently by the same reader after more than 0.3 s, it was assumed to have remained within the adjacent box of the reader. Conversely, when the individual was detected by the same reader within 0.3 s, it was considered to have remained within the pipe where the reader was installed. If an individual was detected by a reader and subsequently by a nonadjacent reader, the individual’s location during the interval was treated as not available (NA). Using this approach, we collected time-series location data for all 102 individuals at 0.1-s intervals over 30 days with 24-hour daily recording.

### Tracking accuracy

The accuracy of this tracking system was assessed by calculating the probability of NA in the time-series location data. This assessment was also performed using the processed data, as described below.

### Annotations of chamber functions

All colonies typically formed a single nest, a toilet, a garbage, and six additional chambers. The nest served as the location where colony members huddled and rested (“nest”; fig. S1A). The toilet was the communal defecation site (“toilet”; fig. S1A), whereas the garbage was where individuals accumulated dirty tissue paper, dried feces, and excess food (“garbage”; fig. S1A).

To examine the spatial stability of three types of functional chambers (nest, toilet, and garbage), we conducted a preliminary observation over 18 weeks using three colonies (*n* = 19, 17, and 26 individuals) housed in the same type of enclosure system used in the main study. We recorded the locations of the functional chambers twice per day. For each chamber type, we calculated the weekly frequency of chamber transitions, defined as the probability that the location of the functional chamber had changed since the previous observation.

In the main dataset used for this study, the time-series location data were extracted at 10-s intervals and restructured. At each time point, the mean number of individuals in each chamber was calculated by averaging across a 6-hour window centered on that time point. In this study, the “nest” was defined as the chamber with the highest mean number of individuals. The locations of “toilet” and “garbage” were manually identified by reviewing video recordings multiple times per day. All remaining locations were categorized as “other.”

### Classification of stay events

A behavioral event (“stay event”) was defined as a period in which an individual remained in a single grid square without transitioning to a different square. The frequency distribution of the duration for each of the four types of stay events was examined, and the maxima and minima were calculated using kernel density estimation. Although the local minimum between the two peaks for nest stay events was 531.1 s, we adopted 600 s as a practical threshold. Stay events in the nest lasting more than 600 s were classified as “Rest,” and those lasting less than 600 s as “Nest” (Fig. 1E). Stay events outside the nest were classified based on their location category (“Toilet,” “Garbage,” and “Other”), regardless of duration. Using these criteria, stay events were classified into five categories: Rest, Nest, Toilet, Garbage, and Other. Stay event data over time were generated by applying these definitions to the time-series location data.

### Processing of time-series data

Because of significant artificial influences, such as feeding and other human care, nearly all individuals in the colony often left the nest simultaneously and became active. To account for this, we defined disturbance phases as periods within continuous phases where the proportion of resting individuals remained below 25% and dropped below 10% at least once. These disturbance phases were excluded from our analyses to minimize the impact of artificial influences.

Previous observations have shown that individuals rest not only in the nest chamber but also in the adjacent tunnels (*20*). In the present study, individuals were often observed remaining in the nest-adjacent tunnels for extended periods. To account for this, we reclassified the stay event as Rest when an individual remained in the nest-adjacent tunnels for more than 600 s.

In some cases, two nests existed simultaneously, primarily during nest transitions. To account for this, we defined a “sub-nest” as a chamber, other than the primary nest, where five or more individuals remained continuously for more than 600 s. Stay events lasting more than 600 s in a sub-nest were also reclassified as Rest. In addition, for data segments with NA values persisting for an unusually long period (more than 30 min), we assumed that the individuals were resting in the nest and reassigned their location to the nest site while reclassifying the stay event as Rest.

### Testing spatial position effects

To assess whether the spatial position of chambers influenced behavior independently of their functional designation, we categorized the nine chambers into three spatial types based on their position: corner, edge, and center. We first examined how functional chambers (nest, toilet, and garbage) were distributed across these spatial types. To isolate the effect of spatial positioning, we focused on “other” chambers and, for each individual and day, calculated the total stay duration per chamber in each spatial category and then derived the ratio of stay duration. To test whether overall behavioral patterns differed by chamber position, we fitted a beta generalized linear mixed model (GLMM) with a logit link function (the `glmmTMB` function from the glmmTMB package in R), with the ratio of total stay duration as the response, chamber position as a fixed effect, and individual and colony as random effects (*n* = 102 individuals × 30 days). To test for caste/sex differences, we analyzed each spatial category separately with beta GLMMs for each category, using caste/sex [breeding female, breeding male, nonbreeding female, and nonbreeding male] as a fixed effect and colony as a random effect (*n*: breeding female = 5 individuals × 30 days; breeding male = 5 × 30; nonbreeding female: 59 × 30; and nonbreeding male: 33 × 30). Post hoc pairwise comparisons were performed on the estimated marginal means (EMMs) with Holm correction (the `emmeans` and `pairs` functions from the emmeans package in R).

### Extraction of parameters for individual behaviors

To identify the behavioral phenotype of each individual, we extracted parameters characterizing individual behavior from 1-day data. For each individual, we calculated the total duration (Sum), the number of events (*N*), and the mean duration (Mean) for each of the five types of stay events. Because Sum and *N* for stay events other than Rest were strongly positively correlated with activity time (total duration of non-Rest stay events), we also included their values relative to activity time (Sum relative and *N* relative) as additional parameters. In total, we extracted 23 parameters describing individual behavior within a 1-day period: Sum (Rest), *N* (Rest), Mean (Rest), Sum (Nest), *N* (Nest), Mean (Nest), Sum relative (Nest), *N* relative (Nest), Sum (Toilet), *N* (Toilet), Mean (Toilet), Sum relative (Toilet), *N* relative (Toilet), Sum (Garbage), *N* (Garbage), Mean (Garbage), Sum relative (Garbage), *N* relative (Garbage), Sum (Other), *N* (Other), Mean (Other), Sum relative (Other), and *N* relative (Other).

### Clustering of behavioral phenotypes

Before analysis, each behavioral parameter was transformed by Box-Cox transformation within each colony, followed by standardization (*z*-transformation) within each colony and day. Using the 23 processed parameters, we clustered 3060 samples (102 individuals × 30 days). To classify individual behavioral phenotypes, we applied a clustering approach based on dimensionality reduction. Specifically, we used UMAP (the `umap` function from the umap package in R) to create a 2D scatter plot. The number of neighbors (n_neighbors) was set to 5, which determines the balance between preserving local and global structures in the embedding. The minimum distance parameter (min_dist) was set to 0.1, the default value, which is widely used in practice and provides a reasonable balance between cluster compactness and preservation of the global data structure. Although we fixed the random seed (random_state = 20), UMAP’s internal stochasticity resulted in nondeterminism. To ensure transparency, we have deposited the original UMAP layout used in the figures. A total of 27 samples, lying far outside the main distribution (fig. S2A), corresponded to the same individual (a nonbreeding male, 7 months old, from colony #4); therefore, all 30 samples from that individual were excluded from the dataset, and UMAP was reapplied to the remaining 101 individuals. For fine-grained clustering, we used a density-based compartmental segmentation approach using the watershed algorithm (the `watershed` function in MATLAB). A density map was generated from the scatter plot using blurring to ensure that the smaller of the two groups was separated as a single independent cluster.

### Characterization of behavioral clusters

To examine behavioral traits associated with each cluster, we compared behavioral parameter values across clusters using linear models (LMs), with parameter value as the response variable and cluster as the explanatory variable (*n* = 3030). Post hoc pairwise comparisons were performed on the EMMs with Holm correction (the `emmeans` and `pairs` functions from the emmeans package in R).

To assess the effects of various individual attributes on behavioral cluster allocation, we constructed eight multinomial logistic regression models (MM1 to MM8) with a logit link function (the `multinom` function from the nnet package in R). The response variable was behavioral cluster (1 to 7), and different individual attributes were included as explanatory variables. An LRT was performed to assess the significance of the explanatory variables for each model. The full dataset (*n* = 3030) was used for models MM1 to MM3, with the following explanatory variables: caste (breeder or nonbreeder) in MM1, body weight (first-order) in MM2, and age (first-order) in MM3. Age was defined as the natural log-transformed postnatal day. A reduced dataset (excluding either breeders or cluster 7; *n* = 2686) was used for models MM4 to MM8, with the following explanatory variables: relative body weight (first- and second-order polynomial) in MM4, relative age (first- and second-order polynomial) in MM5, sex in MM6, sex and relative body weight (first- and second-order polynomial) and their interaction in MM7, and sex and relative age (first- and second-order polynomial) and their interaction in MM8. Relative body weight and relative age were standardized (*z*-transformed) within each colony using the nonbreeder-only dataset.

### Consistency of behavioral phenotypes

To assess the consistency of behavioral phenotypes in individuals, we established a consistency index. This index measured the extent to which an individual’s frequency of assignment to each of the seven behavioral clusters deviated from the expected probability. The consistency index for individual *i* was defined as follows

$$H_{i}=-\;\sum_{k=1}^{K}P_{i}\;\left(k\right)\;\text{ln}P_{i}\;\left(k\right)$$ (1)

$$\text{Consistency index}\;\left(i\right)=1-\frac{H_{i}}{\text{ln}K}$$ (2)

where *P_i_*(*k*) represents the probability of individual *i* being assigned to behavioral cluster *k* over 30 days. *H_i_* denotes the Shannon entropy for individual *i*, quantifying the variability in their cluster assignment across days. *K* is the number of clusters (i.e., *K* = 7). A lower consistency index (close to 0) indicates that an individual was assigned more evenly across clusters, whereas a higher consistency index (close to 1) indicates that an individual was assigned disproportionately to a specific cluster. To statistically evaluate the observed consistency index, we conducted comparisons with randomized datasets. We generated 10,000 sets of simulated data, each consisting of 30 elements, by randomly assigning individuals to clusters 1 to 7 according to the overall mean probability. Ten thousand consistency index values were obtained from these simulated datasets. The *P* value for the observed consistency index was calculated as the proportion of simulated values equal to or more extreme than the observed value. The significance level (α) was set to 0.05.

In addition, we examined colony-level consistency for each behavioral cluster. For each colony and behavioral cluster, we constructed a vector for each day, where the vector length corresponded to the number of individuals, and each element represented whether an individual was assigned to the cluster (1) or not (0). We then calculated cosine similarity between vectors of two consecutive days, yielding 29 cosine similarity values per colony and cluster. To generate a null distribution, we created 100 sets of vectors by randomly shuffling the elements within each vector, resulting in a total of 2900 (29 × 100) cosine similarity values for each colony and cluster. The observed cosine similarity values were compared with the permuted data using the Wilcoxon rank-sum test to assess whether they were significantly higher than the simulated values.

### Within-individual cluster association

To identify strong associations between behavioral clusters, we established a co-assignment index, which quantifies how often two clusters are both assigned within the same individual over time. The co-assignment index for cluster pair (*k*, *l*) was defined as follows

$$C_{\text{emp}}\;\left(k,l\right)=\frac{1}{N}\sum_{i=1}^{N}P_{i}\;\left(k\right)\;P_{i}\;\left(l\right)$$ (3)

$$C_{\text{ref}}\;\left(k,l\right)=\bar{P}\;\left(k\right)\;\bar{P}\;\left(l\right)$$ (4)

$$\begin{array}{c} \text{Co}-\text{assignment index}\;\left(k,l\right)=\frac{C_{\text{emp}}\;\left(k,l\right)}{C_{\text{ref}}\;\left(k,l\right)} \end{array}$$ (5)

Here, $P_{i}\;\left(k\right)$ represents the proportion of days that individual i was assigned to behavioral cluster *k* over the 30-day period, and $\bar{P}\;\left(k\right)$ is the mean assignment probability to cluster *k* across individuals. *N* denotes the total number of individuals (i.e., *N* = 101). $C_{\text{emp}}\;\left(k,l\right)$ captures the average within-individual co-assignment strength for cluster pair (*k*, *l*), and $C_{\text{ref}}\;\left(k,l\right)$ is a baseline co-assignment strength assuming independent assignment. The co-assignment indices greater than 1 indicate that the cluster pair (*k*, *l*) tends to be both assigned within the same individual more frequently than expected under independence.

### Leadership during nest transitions

To evaluate leadership tendency during nest transitions, we quantified the order in which each individual began resting in the newly designated nest after each transition event. For each nest transition event, we identified the timing at which each individual first initiated a rest episode in the newly designated nest, within a time window from 2 hours before to 12 hours after the nest location changed. The order of rest initiation across individuals was recorded for each event. For analysis, we included only those nest transitions in which more than 80% of the colony members began resting in the new nest within this window. A total of 99 events were analyzed across the five colonies (colony #1: 23 events; #2: 33; #3: 21; #4: 7; and #5: 15).

To test whether certain individuals consistently initiated rest earlier or later (i.e., exhibited strong leadership tendencies), we fitted a Bayesian ordinal GLMM with a logit link function (the `brm` function from the brms package in R). The response variable was the rank order of rest initiation, with colony as a fixed effect and individual identity as a random effect (total *n* = 1995 observations). Default weakly informative priors were used for all parameters. Each model was run with four Markov chains and 4000 iterations per chain (including 1000 warm-up iterations). Convergence was assessed based on Rhat values (<1.01) and inspection of trace plots. To assess individual differences in nest transition leadership, we extracted the posterior estimates of the individual-level random effects and computed summary statistics including the posterior mean and 95, 99, and 99.9% credible intervals. Significance was determined based on whether these intervals excluded zero (group-level mean).

To test whether leadership tendencies differed among behavioral clusters, we fitted another ordinal GLMM under the same modeling conditions, using the same response variable, with behavioral cluster as a fixed effect and colony as a random effect (*n*: cluster 1 = 313; cluster 2 = 246; cluster 3 = 192; cluster 4 = 232; cluster 5 = 209; cluster 6 = 530; and cluster 7 = 206). Post hoc pairwise comparisons were performed on the estimated latent means with Holm correction (the `emmeans` and `pairs` functions from the emmeans package in R).

### Quantification of activity rhythm synchrony

The data on whether each individual was in a “Rest” or “Active” (i.e., non-Rest) state every 0.1 s over the 30-day period were derived from the time-series stay event data. These data were then extracted at 10-s intervals and restructured to generate a binary vector for each individual. For each dyad within a colony, *N*_Synchrony_ was defined as the total number of time points in which both individuals simultaneously exhibited the same state.

To generate randomized surrogate data, 9999 surrogate vectors were created for each individual by randomly permuting the elements while preserving the cyclic sequence structure. *N*_Synchrony_ was then computed between the actual vector of one individual and each permuted vector of the other, resulting in 9999 simulated *N*_Synchrony_ values per dyad.

### Quantification of spatial proximity

The data on the location of each individual within a five-by-five grid every 0.1 s over the 30-day period, with “nest” indicated when the individual was in the nest, were derived from the time-series location and stay event data. These data were then extracted at 10-s intervals and restructured to generate a location vector for each individual. For each dyad within a colony, *N*_Proximity_ was defined as the total number of time points when both individuals were simultaneously in the same grid square outside the nest. For each dyad, the time points when both individuals were simultaneously outside the nest were extracted, and their respective vectors were reconstructed using only these time points.

To generate randomized surrogate data, 9999 surrogate vectors were created for one individual from a dyad by randomly permuting the elements while preserving the cyclic sequence structure. *N*_Proximity_ was then computed between the actual vector of one individual and each permuted vector of the other, resulting in 9999 simulated *N*_Proximity_ values per dyad.

### Quantification of follow behavior

Movement event data were derived from time-series location data collected over the 30-day period, identifying the timing and trajectory of individual movements within a five-by-five grid, where each movement corresponded to a transition between adjacent grid cells. Based on these data, two vectors were constructed for each individual: one representing the timing of movement events and the other representing the movement trajectory. For each dyad within a colony, a follow behavior event was recorded when one individual (A) executed a specific movement (i.e., a transition between adjacent two grid cells), and another individual (B) performed the same movement within a 3-s window. Follow behavior events were recorded separately for both directions: A following B and B following A. The total number of follow behavior events for each directed dyad was defined as *N*_Follow_.

To generate randomized surrogate data, 999 surrogate vector sets were created for each individual. The movement timing vector was preserved, whereas the movement trajectory vector was reconstructed by randomly permuting the elements while preserving the cyclic sequence structure. *N*_Follow_ was then computed for each directed dyad between the actual movement vector set of one individual and the randomized movement vector set of the other, resulting in 999 simulated *N*_Follow_ values per directed dyad.

### Calculation of statistical significance indicators in social relationship indices

The *P* value for each (directed) dyad was determined by ranking the actual value within the distribution of 10,000 (*N*_Synchrony_ and *N*_Proximity_) or 1000 (*N*_Follow_) values, including the actual value and corresponding simulated values. The *P* value was calculated as the proportion of values equal to or more extreme than the actual value. If the actual value ranked within the top or bottom 2.5% of the distribution (*P* < 0.05), the (directed) dyad was considered significantly synchronous, proximal, or exhibiting significantly frequent follow behavior for the top 2.5%, or significantly asynchronous, distal, or exhibiting significantly infrequent follow behavior for the bottom 2.5%. Otherwise, it was considered nonsignificant.

For further statistical analyses, synchrony index, proximity index, and follow index were calculated for each (directed) dyad by standardizing the actual values within the distribution of simulated values using the formula

$$\text{Index}=\frac{\text{Actual value}-\text{Mean of simulated values}}{\text{Standard deviation of simulated value}}$$ (6)

The follow index was transformed using the Yeo-Johnson transformation and standardization (*z*-transformation) across all directed dyads, considering its distribution structure.

Pairwise matrix data for synchrony, proximity, and follow index, along with corresponding *P* value, were generated and visualized for each colony (the `pheatmap` function from the pheatmap package in R). The matrices for synchrony, proximity, and follow index were displayed alongside dendrograms constructed by hierarchical clustering with Ward’s method. For each colony, (directed) dyads with significantly synchronous, proximal, or frequent follow behavior were extracted to construct undirected (synchrony and proximity) or directed (follow behavior) weighted networks (the `graph_from_data_frame` function from the igraph package in R). The index (synchrony, proximity, or follow index) was used as the edge weight.

### Testing the effects of attribute factors on the social relationships

To examine the effects of various individual attributes on each index of the social relationships, we performed statistical analyses including 17 linear (mixed) models (LM_S1–5, LM_P1–5, and LM_F1–7) with synchrony, proximity, or follow index as a response variable and attribute factors as explanatory variables. The linear mixed models (LMMs) included attribute factors as fixed effects and colony as a random effect (the `lmer` function from the lmerTest package in R). The following indices were used as the response variable: synchrony index for LM_S1–5, proximity index for LM_P1–5, and follow index for LM_F1–7. LRT was performed to assess the significance of the fixed effects in each model.

Statistical analyses on the synchrony index and proximity index were conducted as follows. To examine the overall colony level for each index, LMs (LM_S1 and LM_P1) were fitted with colony as the explanatory variable using the full dataset (*n* = 1000). For each colony, the EMM was compared to zero (representing the shuffling control) using a one-sample *t* test (the `emmeans` and `test` functions from the emmeans package in R). To investigate differences by caste combination (B-B: breeder-breeder, B-NB: breeder-nonbreeder, and NB-NB: nonbreeder-nonbreeder), LMMs (LM_S2 and LM_P2) were fitted with caste combination as the fixed effect using the full dataset (*n* = 1000). To examine differences by sex combination in B-NB dyads (*n* = 184), LMMs (LM_S3 and LM_P3) were fitted with sex combination as the fixed effect. Similarly, for NB-NB dyads (*n* = 811), LMMs (LM_S4 and LM_P4) were fitted with sex combination as the fixed effect. For the models LM_S2–4 and LM_P2–4, pairwise comparisons were subsequently conducted using the EMMs with Holm correction (the `emmeans` and `pairs` functions from the emmeans package in R). To examine the level of each behavioral cluster combination, LMMs (LM_S5 and LM_P5) were fitted with the synchrony index or proximity index as the response and the combination of behavioral clusters as the fixed effect, using a dataset that excluded dyads with one unclustered individual (*n* = 979). For each cluster combination, the EMM was compared to the observed mean using a one-sample *t* test. In the models, each individual’s behavioral cluster was assigned as the most frequent cluster over the 30-day period. If multiple clusters were equally the most frequent, the cluster with the lowest overall occurrence across all individuals within those clusters was selected as the representative cluster. Last, the relationship between the synchrony index and the proximity index was examined using a Pearson correlation test, both across all colonies combined and separately within each colony.

Statistical analyses on the follow index were conducted as follows. Here, directed dyads are denoted as X-Y, where X represents the follower and Y the followee (e.g., B-NB: a breeder followed a nonbreeder). To examine differences by caste pair (B-B, B-NB, NB-B, and NB-NB), an LMM (LM_F1) was fitted with the follow index as the response and caste pair as the fixed effect, using the full dataset (*n* = 2000). For each caste pair, the EMM was compared to the shuffling control using a one-sample *t* test. To examine sex differences within B-B dyads, paired *t* tests were performed comparing breeding female and breeding male (*n* = 5 pairs). To examine differences among sex pairs in other dyads, LMMs were fitted with sex pair as the fixed effect for B-NB dyads (LM_F2, *n* = 184), NB-B dyads (LM_F3, *n* = 184), and NB-NB dyads (LM_F4, *n* = 1622), respectively. For the models LM_F1–4, pairwise comparisons were subsequently conducted using the EMMs with Holm correction. To investigate factors contributing to the differences in how breeding females and breeding males follow nonbreeders, two LMMs were fitted with breeder sex, nonbreeder’s relative body weight (first- and second-order polynomial, LM_F5) or relative age (first- and second-order polynomial, LM_F6), and their interaction as the fixed effects, using the B-NB dataset (*n* = 184). To examine the level of each behavioral cluster pair, an LMM (LM_F7) was fitted with cluster pair as the fixed effect using a dataset that excluded dyads with one unclustered individual (*n* = 1958). For each cluster pair, the EMM was compared to zero (representing the observed mean) using a one-sample *t* test.

### Network robustness across time

To assess the temporal robustness of the derived networks (synchrony, proximity, and follow), we divided the 30-day dataset into six nonoverlapping 5-day subsets. For each subset, we repeated the same procedures as described to compute the synchrony, proximity, or follow indices in the corresponding sections above. Notably, although 9999 surrogate datasets were generated for synchrony, only 999 were generated for proximity and follow due to data size constraints.

To examine whether certain individuals consistently occupied central positions within each type of social network, we calculated strength centrality for each individual within each 5-day subset. Strength centrality was defined as the sum of the weights of all edges connected to each individual, where edge weights corresponded to synchrony, proximity, or follow indices. For the directed following network, we calculated in-strength and out-strength separately, representing the sum of follow indices when acting as the followee (being followed) and when acting as the follower (following others), respectively. We fitted Bayesian LMMs (the `brm` function from the brms package in R), with strength centrality as a response, colony-subset combination as a fixed effect, and individual identity as a random effect (total *n* = 102 individuals × 6 subsets). Default weakly informative priors were used for all parameters. Each model was run with four Markov chains and 4000 iterations per chain (including 1000 warm-up iterations). Convergence was assessed based on Rhat values (<1.01) and inspection of trace plots. We then obtained posterior estimates for each individual and computed summary statistics including the posterior mean and 95, 99, and 99.9% credible intervals. Significance was determined based on whether these intervals excluded zero (group-level mean).

To examine whether specific behavioral clusters play central roles in each network, we fitted LMMs (the `lmer` function from the lmerTest package in R) with strength centrality as a response, behavioral cluster as a fixed effect, and individual identity and colony-subset combination as random effects (*n* per subset: cluster 1 = 16 individuals; cluster 2 = 14; cluster 3 = 11; cluster 4 = 13; cluster 5 = 7; cluster 6 = 31; and cluster 7 = 9). For each cluster, the EMM was compared to zero (representing the overall mean) using a one-sample *t* test (the `emmeans` and `test` functions from the emmeans package in R).

### Video-based behavioral annotation of follow events

To complement the automated tracking data and assess the behavioral context of follow events, we conducted a manual video-based classification of social interactions. For each of the four breeder/nonbreeder dyad classes (B-B, B-NB, NB-B, and NB-NB), we randomly sampled 100 follow events from the full dataset (number of dyads: B-B = 10; B-NB = 184; NB-B = 184; and NB-NB = 5742) (total number of events: B-B = 9182; B-NB = 41,264; NB-B = 28,345; and NB-NB = 223,976). Each sampled follow event was linked to the corresponding time window in the video recordings and manually annotated into one of four interaction categories: (i) affiliative (e.g., mating, anogenital nuzzling, and play fighting), (ii) agonistic (e.g., shoving and biting), (iii) tail tugging (defined as one individual pulling the tail of another, typically with the tail-puller designated as the followee), and (iv) neutral (no discernible interaction). Representative examples of each interaction type are provided in supplementary movies S1 to S6.

### Software and computational tools

The algorithm for conversion from the RFID detection data to location data was performed in Python 3.10 using NumPy and pandas. The watershed algorithm was performed in MATLAB (R2022a, MathWorks, USA), whereas all other data processing, analyses, and visualization were conducted in R (version 4.2.3, R Core Team, 2023). All statistical results are summarized in table S1 (submitted as a separate Excel file).
