# Supplementary material for: Quantitative and systematic behavioral profiling reveals social complexity in eusocial naked mole-rats
Source: Sci Adv. 2025 Oct 8;11(41):eady0481. doi: 10.1126/sciadv.ady0481 (PMC12507003; doi:10.1126/sciadv.ady0481)
Supplement: Supplementary file 1 — Figs. S1 to S8 Legends for movies S1 to S7 Legend for table S1 [file sciadv.ady0481_sm.pdf]

Science Advances
AAAS

Supplementary Materials for

Quantitative and systematic behavioral profiling reveals social complexity in eusocial naked mole-rats

Masanori Yamakawa *et al.*

Corresponding author: Teruhiro Okuyama, okuyama@iqb.u-tokyo.ac.jp;
Kyoko Miura, miura.kyoko.314@m.kyushu-u.ac.jp; Masanori Yamakawa, yamakawamanori1008@gmail.com

Sci. Adv. **11**, eady0481 (2025)
DOI: 10.1126/sciadv.ady0481

The PDF file includes:

Figs. S1 to S8
Legends for movies S1 to S7
Legend for table S1

Other Supplementary Material for this manuscript includes the following:

Movies S1 to S7
Table S1

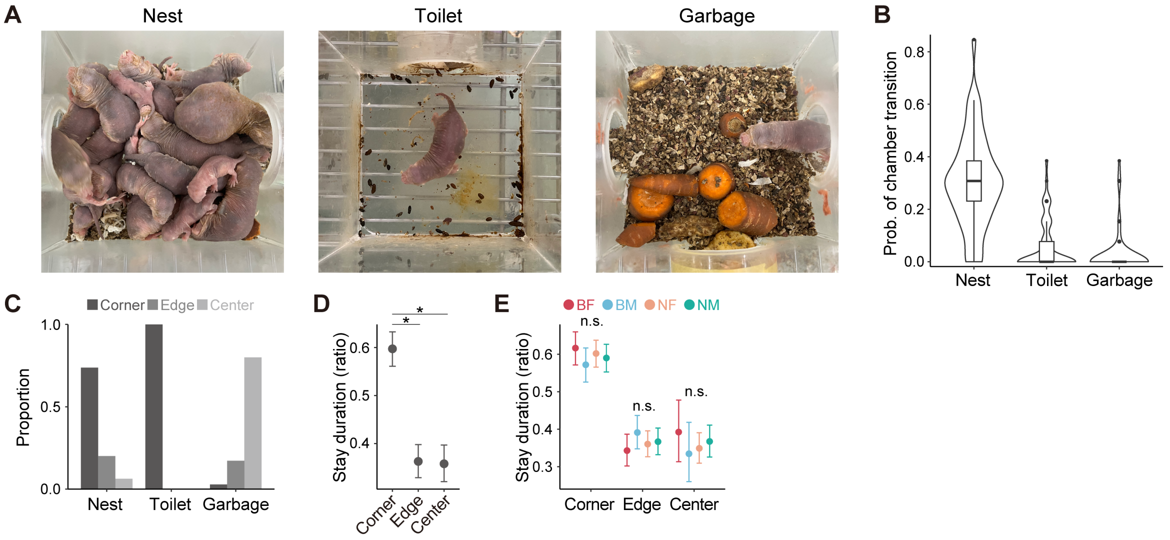

Fig. S1. Functional use of chambers and spatial organization in naked mole-rat colonies. (**A**) Photographs of functional chambers observed in naked mole-rat colonies, including nest (left), toilet (center), and garbage chamber (right). (**B**) Weekly probability of chamber transition for each chamber type, based on preliminary observations conducted over 18 weeks (nest and toilet) or 11 weeks (garbage). Data are shown as violin plots overlaid with box plots. (**C**) Proportion of chamber positions (corner, edge, center) across each functional chamber type. (**D**) Differences in the ratio of stay duration among chamber positions within "other" chambers. Significance was determined based on Holm-adjusted pairwise comparisons of EMMs. n = 102 individuals × 30 days. *$p < 0.05$. (**E**) Caste/sex differences in the ratio of stay duration in each chamber position within "other" chambers. Significance was determined based on Holm-adjusted pairwise comparisons of EMMs. n: BF = 5 individuals × 30 days; BM = 5 × 30; NF: 59 × 30; NM: 33 × 30. n.s.: Non-significant across all pairwise comparisons.

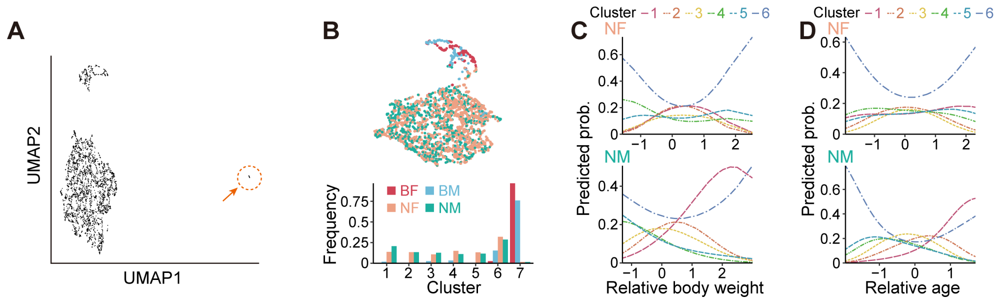

Fig. S2. Supporting and additional analyses of behavioral phenotypes (related to Fig. 2). (**A**) UMAP two-dimensional scatter plot of the full dataset (*n* = 3,060). A red arrow and dashed circle represent 27 outlier samples from a single individual, separated from the main distribution. All samples from this individual (*n* = 30) were excluded from subsequent analyses. (**B**) Relationship between behavioral cluster assignment and sex, based on the same dataset used in Fig. 2 (*n* = 3,030). The UMAP scatter plot from Fig. 2B is shown with individual points colored by sex class (BF, BM, NF, NM). Lower panels show bar plots representing the frequency of cluster assignments for each sex. BF: breeding female; BM: breeding male; NF: nonbreeding female; NM: nonbreeding male. (**C** and **D**) Relationships between predicted probabilities of cluster assignment and relative body weight (C) or relative age (D), shown separately for nonbreeding females and nonbreeding males. Curves represent predictions from multinomial models including an interaction between sex and the respective predictor. The models were fitted to nonbreeder samples assigned to clusters 1–6 (*n* = 2,686).

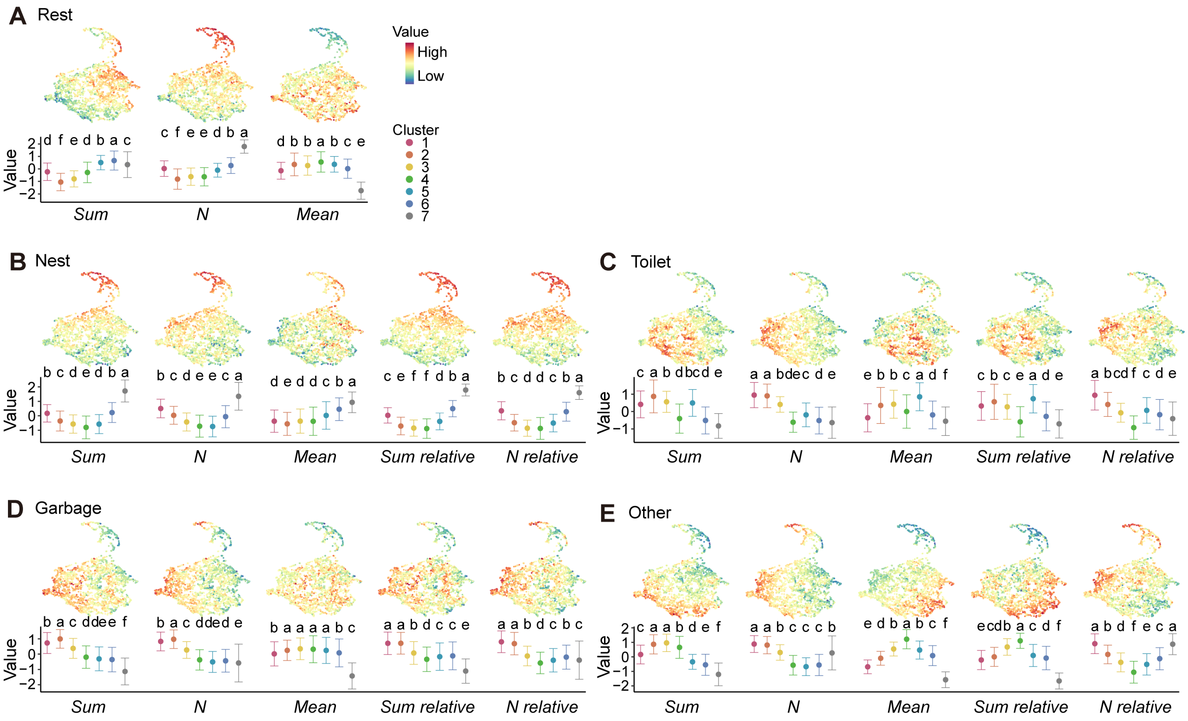

Fig. S3. Differences among behavioral clusters across all 23 measured parameters. (**A** to **E**) Comparisons across behavioral clusters for all 23 parameters, grouped by stay event type. (A) Rest-related parameters (3), (B) Nest-related (5), (C) Toilet-related (5), (D) Garbage-related (5), and (E) Other-related (5). For each parameter, the upper panel shows individual values overlaid on the UMAP scatter plot, and the lower panel displays cluster-wise means (dots) with SD (error bars), as in Fig. 2, D to G. Different letters indicate significant differences between clusters, based on Holm-adjusted pairwise comparisons of EMMs.

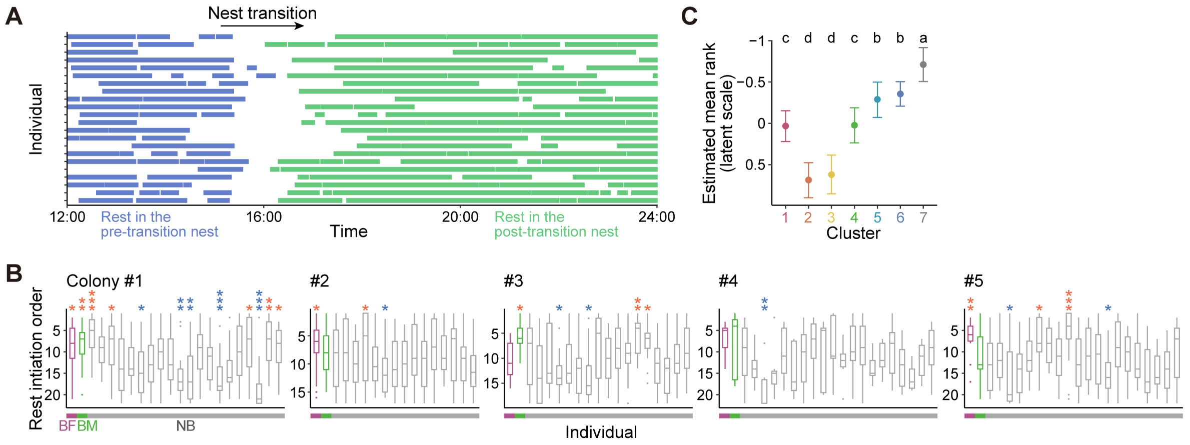

Fig. S4. Individual and cluster-level differences in leadership during collective nest transitions. (**A**) Example of a nest transition event. Time-series plot shows when and where each individual in colony #1 was resting over a 12-hour period. Colors correspond to chamber IDs in Fig. 1B. (**B**) Individual differences in rest initiation order. Data are shown as boxplots. n = 1,995 in total. Individuals are arranged in the same order as in Fig. 3, A to C. Significance was defined as exclusion of group-level mean from the credible interval of the individual-level random effect estimate in the Bayesian ordinal GLMM, at the 95% (*), 99% (**), or 99.9% (***) level. Red and blue asterisks denote significantly higher and lower leadership tendencies, respectively. (**C**) Estimated latent leadership scores from the ordinal GLMM for each behavioral cluster. Points represent posterior means, and error bars represent 95% credible intervals. n: cluster 1 = 313, cluster 2 = 246, cluster 3 = 192, cluster 4 = 232, cluster 5 = 209, cluster 6 = 530, cluster 7 = 206. Different letters indicate significant differences based on Holm-adjusted pairwise comparisons of estimated latent means.

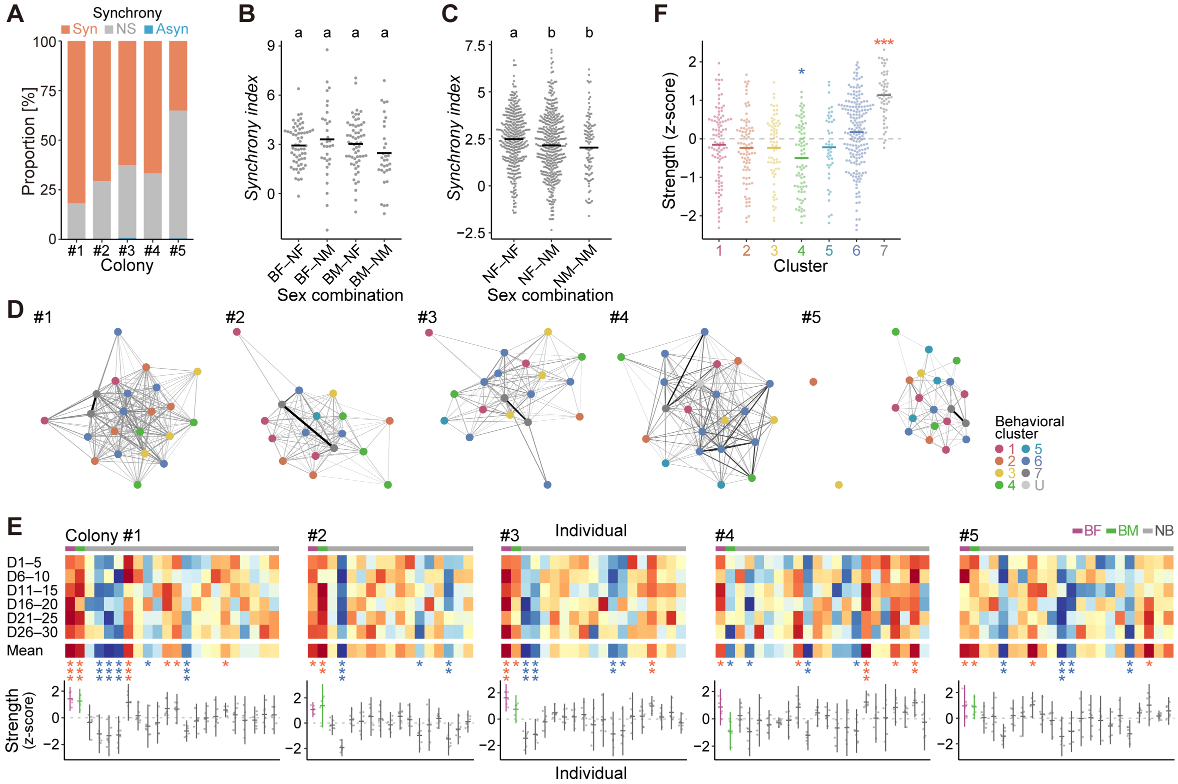

Fig. S5. Additional analyses of activity rhythm synchrony (related to Fig. 4). (**A**) Proportions of dyads that were significantly synchronous (Syn), asynchronous (Asyn), or non-significant (NS) in activity rhythms for each colony. (**B** and **C**) Differences in synchrony index among sex combinations within breeder–nonbreeder (B) and nonbreeder–nonbreeder (C) dyads. BF: breeding female; BM: breeding male; NF: nonbreeding female; NM: nonbreeding male. Bars represent estimates from LMMs. *n* for (B): BF–NF = 60; BF–NM = 32; BM–NF = 60; BM–NM = 32. *n* for (C): NF–NF = 332; NF–NM = 383; NM–NM = 96. Different letters indicate significant differences based on Holm-adjusted pairwise comparisons of EMMs. (**D**) Same network of significantly synchronous dyads as in Fig. 4E, with node colors indicating the behavioral cluster of each individual (U: un-clustered). (**E**) Individual differences and consistency in synchrony strength centralities. Heatmaps show strength values across each 5-day subset and mean for each individual. Scatter plots show mean strength and SD for each individual. *n* = 102 individuals × 6 subsets. Dashed lines represent zero (group-level mean). Individuals are arranged in the same order as in Fig. 3, A to C. Strength values were Yeo–Johnson transformed and z-scored for visualization. Significance was defined as exclusion of group-level mean from the credible interval of the

individual-level random effect estimate in the Bayesian LMM, at the 95% (*), 99% (**), or 99.9% (***) level. Red and blue asterisks denote significantly higher and lower strengths, respectively. (**F**) Synchrony strengths for each behavioral cluster, compared to the overall mean (dashed line) using one-sample *t*-tests on EMMs. *n* per subset: cluster 1 = 16; cluster 2 = 14; cluster 3 = 11; cluster 4 = 13; cluster 5 = 7; cluster 6 = 31; cluster 7 = 9. Bars represent mean strengths. Red and blue asterisks denote significantly higher and lower strengths, respectively. *$p < 0.05$, **$p < 0.01$, ***$p < 0.001$.

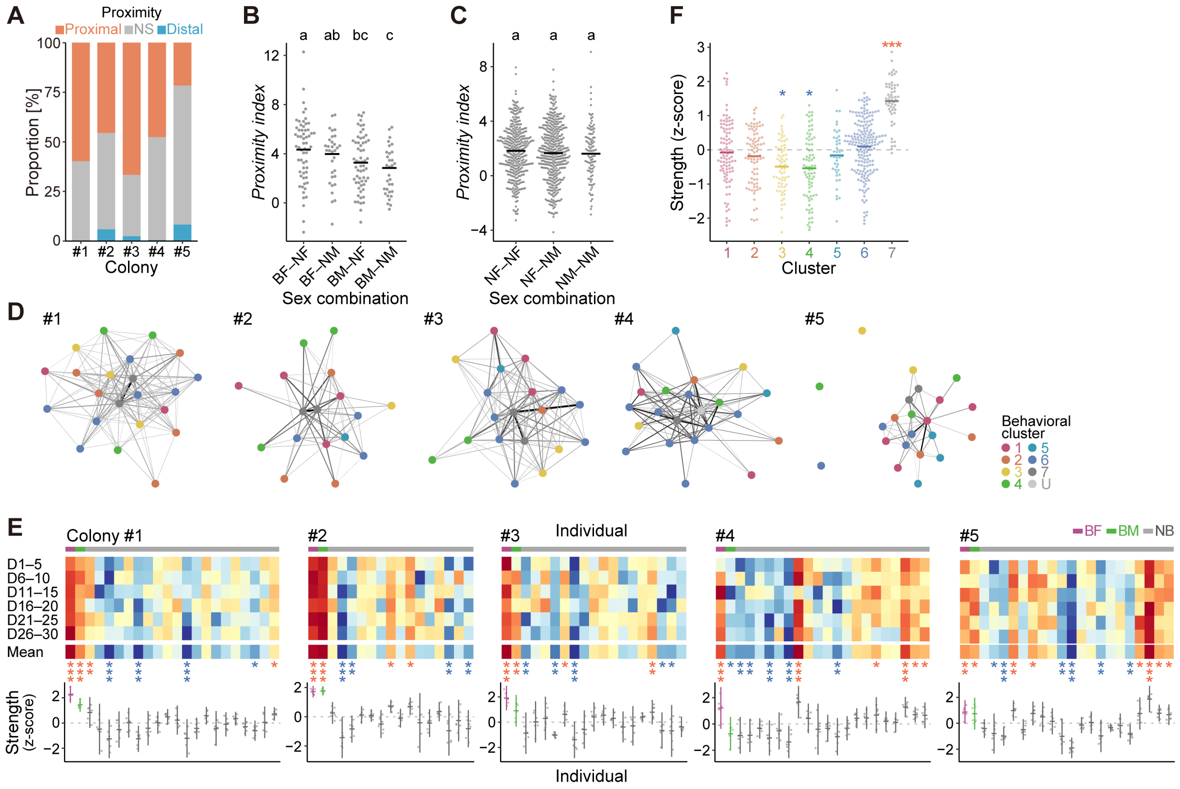

Fig. S6. Additional analyses of spatial proximity (related to Figure 5). (**A**) Proportions of dyads that were significantly proximal, distal, or non-significant (NS) in activity rhythms for each colony. (**B** and **C**) Differences in proximity index among sex combinations within breeder–nonbreeder (B) and nonbreeder–nonbreeder (C) dyads. BF: breeding female; BM: breeding male; NF: nonbreeding female; NM: nonbreeding male. Bars represent estimates from LMMs. *n* for (B): BF–NF = 60; BF–NM = 32; BM–NF = 60; BM–NM = 32. *n* for (C): NF–NF = 332; NF–NM = 383; NM–NM = 96. Different letters indicate significant differences based on Holm-adjusted pairwise comparisons of EMMs. (**D**) Same network of significantly proximal dyads as in Fig. 5E, with node colors indicating the behavioral cluster of each individual (U: un-clustered). (**E**) Individual differences and consistency in proximity strength centralities. Heatmaps show strength values across each 5-day subset and mean for each individual. Scatter plots show mean strength and SD for each individual. *n* = 102 individuals × 6 subsets. Dashed lines represent zero (group-level mean). Individuals are arranged in the same order as in Fig. 3, A to C. Strength values were Yeo–Johnson transformed and z-scored for visualization. Significance was defined as exclusion of group-level mean from the credible interval of the individual-level random effect estimate in the

Bayesian LMM, at the 95% (*), 99% (**), or 99.9% (***) level. Red and blue asterisks denote significantly higher and lower strengths, respectively. (**F**) Proximity strengths for each behavioral cluster, compared to the overall mean (dashed line) using one-sample *t*-tests on EMMs. *n* per subset: cluster 1 = 16; cluster 2 = 14; cluster 3 = 11; cluster 4 = 13; cluster 5 = 7; cluster 6 = 31; cluster 7 = 9. Bars represent mean strengths. Red and blue asterisks denote significantly higher and lower strengths, respectively. *$p < 0.05$, **$p < 0.01$, ***$p < 0.001$.

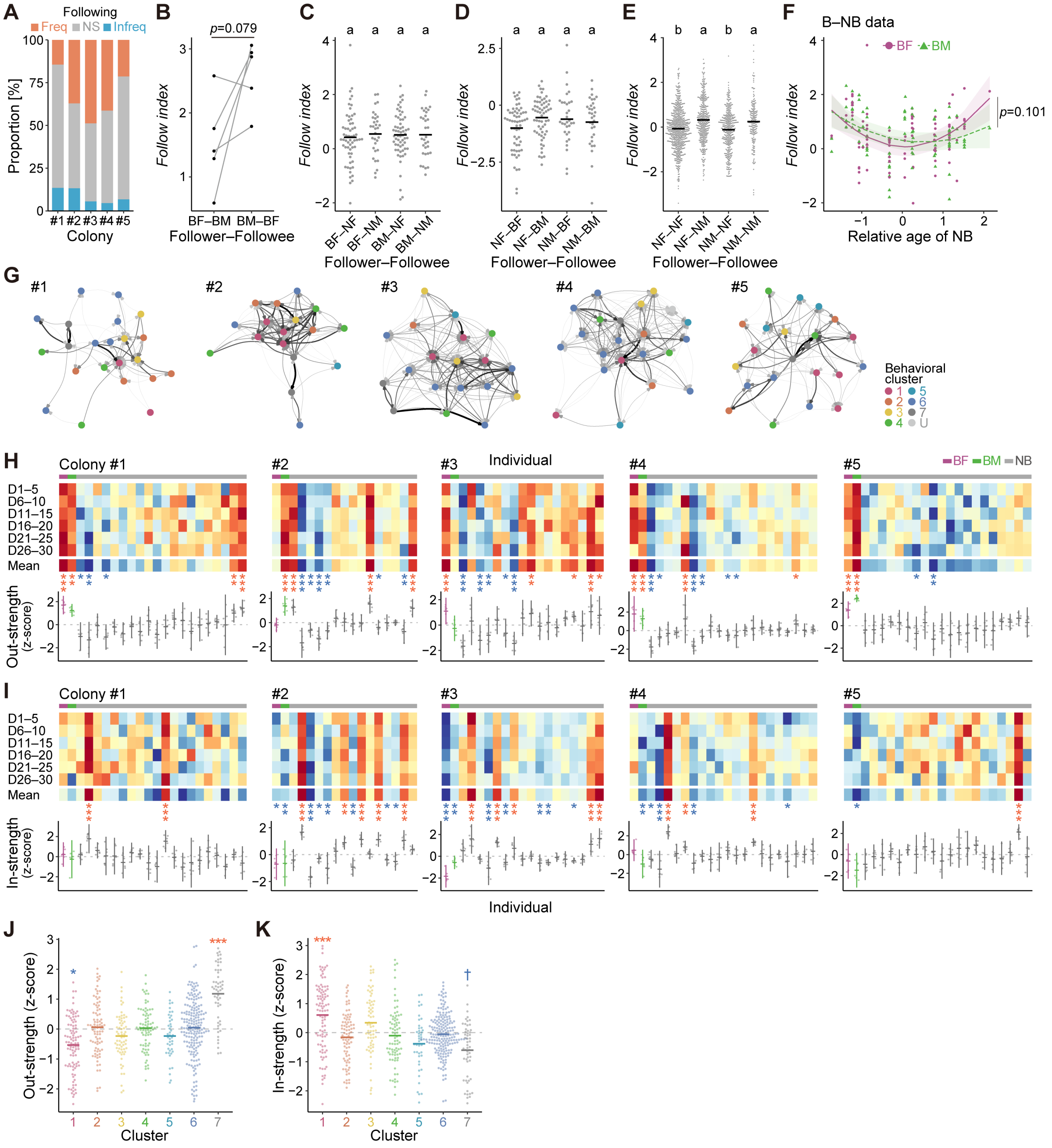

Fig. S7. Additional analyses of following relationships (related to Fig. 6). (**A**) Proportions of directed dyads that showed significantly more frequent (Freq), less frequent (Infreq), or non-significant (NS) following for each colony. (**B** to **E**) Differences in follow index among sex pairs within breeder–breeder (B), breeder–nonbreeder (C), nonbreeder–breeder (D), and nonbreeder–nonbreeder (E) dyads. BF: breeding female; BM: breeding male; NF: nonbreeding female; NM: nonbreeding male. Significant difference was tested using a paired t-test for (B). Bars represent

estimates from LMMs. Different letters indicate significant differences based on Holm-adjusted pairwise comparisons of EMMs. *n* for (B): 5 sex pairs; *n* for (C): BF–NF = 60, BF–NM = 32, BM–NF = 60, BM–NM = 32; *n* for (D): NF–BF = 60, NM–BF = 32, NF–BM = 60, NM–BM = 32; *n* for (E): NF–NF = 664, NF–NM = 383, NM–NF = 383, NM–NM = 192. (**F**) Relationship between follow indices and nonbreeder's relative age across breeder sex, based on the B–NB dataset. Curves represent group-level predictions from an LMM (n = 92 per group). The *p-value* reflects the significance of an interaction between breeder sex and nonbreeder's relative age. (**G**) Same network as in Fig. 6E, consisting of directed dyads that showed significantly more frequent following, with node colors indicating the behavioral cluster of each individual (U: un-clustered). (**H** and **I**) Individual differences and consistency in out-strength (H) and in-strength (I) centralities. Heatmaps show strength values across each 5-day subset and mean for each individual. Scatter plots show mean strength and SD for each individual. n = 102 individuals × 6 subsets. Dashed lines represent zero (group-level mean). Individuals are arranged in the same order as in Fig. 3, A to C. Strength values were Yeo–Johnson transformed and z-scored for visualization. Significance was defined as exclusion of group-level mean from the credible interval of the individual-level random effect estimate in the Bayesian LMM, at the 95% (*), 99% (**), or 99.9% (***) level. Red and blue asterisks denote significantly higher and lower strengths, respectively. (**J** and **K**) Out-strengths (J) and in-strengths (K) for each behavioral cluster, compared to the overall mean (dashed line) using one-sample *t*-tests on EMMs. *n* per subset: cluster 1 = 16; cluster 2 = 14; cluster 3 = 11; cluster 4 = 13; cluster 5 = 7; cluster 6 = 31; cluster 7 = 9. Bars represent mean strengths. Red and blue asterisks denote significantly higher and lower strengths, respectively. †$p < 0.1$, *$p < 0.05$, **$p < 0.01$, ***$p < 0.001$.

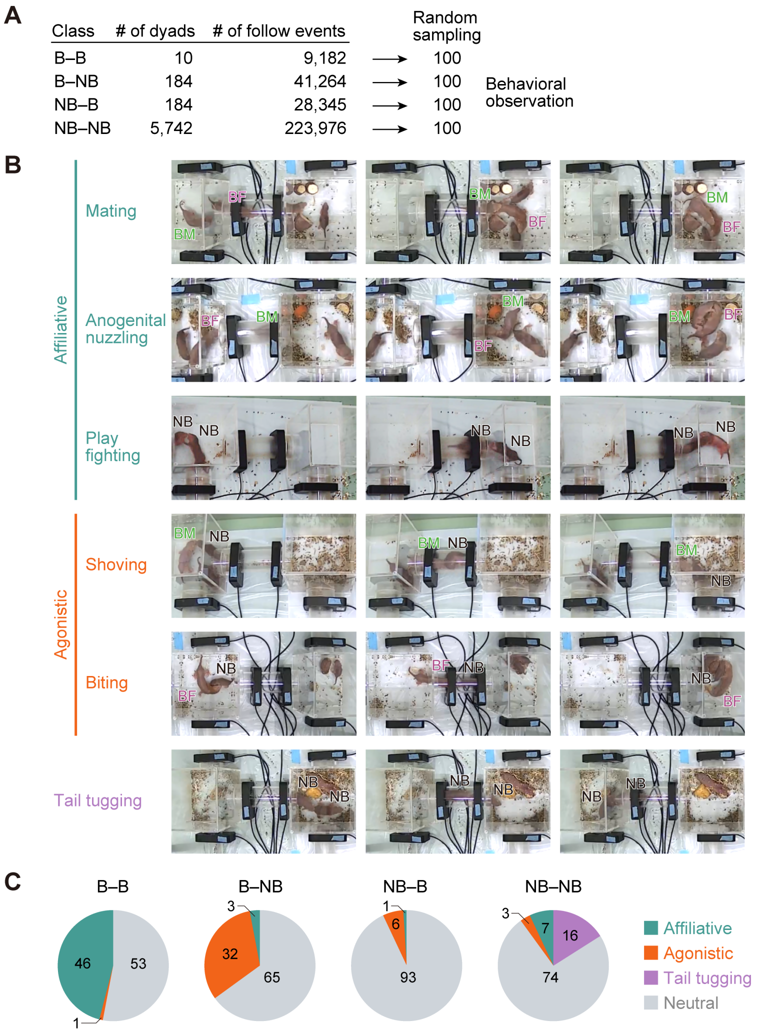

Fig. S8. Video-based analysis of interaction types during follow events (related to Fig. 6). (**A**) Procedure for selecting follow events for behavioral annotation. From each breeder/nonbreeder dyad class (B–B, B–NB, NB–B, NB–NB), 100 follow events were randomly selected for manual classification based on video footage. (**B**) Classification of interaction types in follow events. Each selected event was categorized into one of four types: affiliative (e.g., mating, anogenital nuzzling, play fighting), agonistic (e.g., shoving, biting), tail tugging (typically with the tail-puller recorded as the followee), or neutral (no discernible interaction). Representative examples of the observed interaction types are shown in movies S1 to S6. (**C**) Proportions of interaction types across dyad classes. Pie charts show the distribution of interaction types within each dyad class (n = 100 events per class).

Movie S1.

Mating observed between two breeders around a follow event.

Movie S2.

Anogenital nuzzling between two breeders around a follow event.

Movie S3.

Play fighting between two nonbreeders around a follow event.

Movie S4.

A breeding male shoves a nonbreeder around a follow event in which the breeder is the follower.

Movie S5.

A breeding female bites a nonbreeder around a follow event in which the breeder is the follower.

Movie S6.

Tail tugging between two nonbreeders around a follow event.

Movie S7.

Time-lapse video showing the entire naked mole-rat colony within the housing system, recorded over 30 minutes and accelerated 50-fold.

Table S1. Summary of all statistical results. (separate file)

This Excel file contains detailed results of all statistical analyses presented in the main text and supplementary materials, including model specifications, sample sizes, test statistics, and p-values. Each sheet corresponds to a specific figure described in the paper.
